# Recent advances in modified biochars for the adsorptive removal of typical antibiotics from water: a comparative review of performance, influencing factors, and underlying mechanisms

**DOI:** 10.1039/d6ra06945b

**Published:** 2026-09-01

**Authors:** Pengxuan Ding, Juanjuan Lu, Lianjun Xu, Qi Zhou, Min Jiang, Zhili Yan, Pinhong Yang

**Affiliations:** a Key Laboratory of Featured Aquaculture, College of Agriculture Forestry Science and Technology, Hunan Applied Technology College Changde 415100 China 2252105118@qq.com yph588@163.com

## Abstract

Antibiotic contamination in aquatic environments has raised increasing concern because conventional treatment processes often show incomplete removal of ionizable antibiotics. Modified biochar has emerged as a promising adsorbent, but its performance varies substantially with antibiotic class, modification route, and solution chemistry. This review provides a focused, class-resolved and quantitative comparison of modified biochars for the adsorption of tetracyclines (TCs), sulfonamides (SAs), and fluoroquinolones (FQs) across five major modification strategies: alkali activation, acid and salt activation, metal and magnetic composites, heteroatom doping, and hybrid advanced composites. Based on 50 retained primary publications, reported maximum adsorption capacities (*Q*_max_) are interpreted as performance distributions rather than absolute rankings across heterogeneous experimental conditions. *Q*_max_ ranged from 77.60–2242.00 mg g^−1^ for TCs, 4.18–1083.00 mg g^−1^ for SAs, and 11.62–1295.40 mg g^−1^ for FQs. TCs showed the highest upper-range capacities because their polycyclic structures and multiple functional groups favor multipoint interactions, whereas SAs exhibited greater variability because of pH-sensitive ionization and weaker complexation ability; FQ adsorption was strongly dependent on surface electronic structure, polarity, and pH-regulated speciation. Across systems, adsorption performance reflects the coupling of hierarchical pore accessibility, surface charge, aromatic carbon domains, heteroatom-derived active sites, and metal coordination centers. These interactions are organized into a progressive interfacial framework in which pore accessibility enables retention, electrostatic interactions regulate molecular approach and orientation, π–π electron donor–acceptor interactions and hydrogen bonding provide molecular recognition and stabilization, and surface coordination contributes site-specific anchoring. Importantly, high *Q*_max_ alone does not indicate application readiness. Real-water matrix resistance, regeneration stability, leaching risk, adsorbent recovery, continuous-flow operation, preparation complexity, techno-economic feasibility, and life-cycle impacts should therefore be jointly considered when evaluating modified biochars for antibiotic-contaminated water treatment.

## Abstract 2

Modified biochars remove TCs, SAs and FQs through a progressive interfacial pathway integrating pore accessibility, charge-regulated molecular approach, molecular recognition and site-specific anchoring.

## Graphical abstract

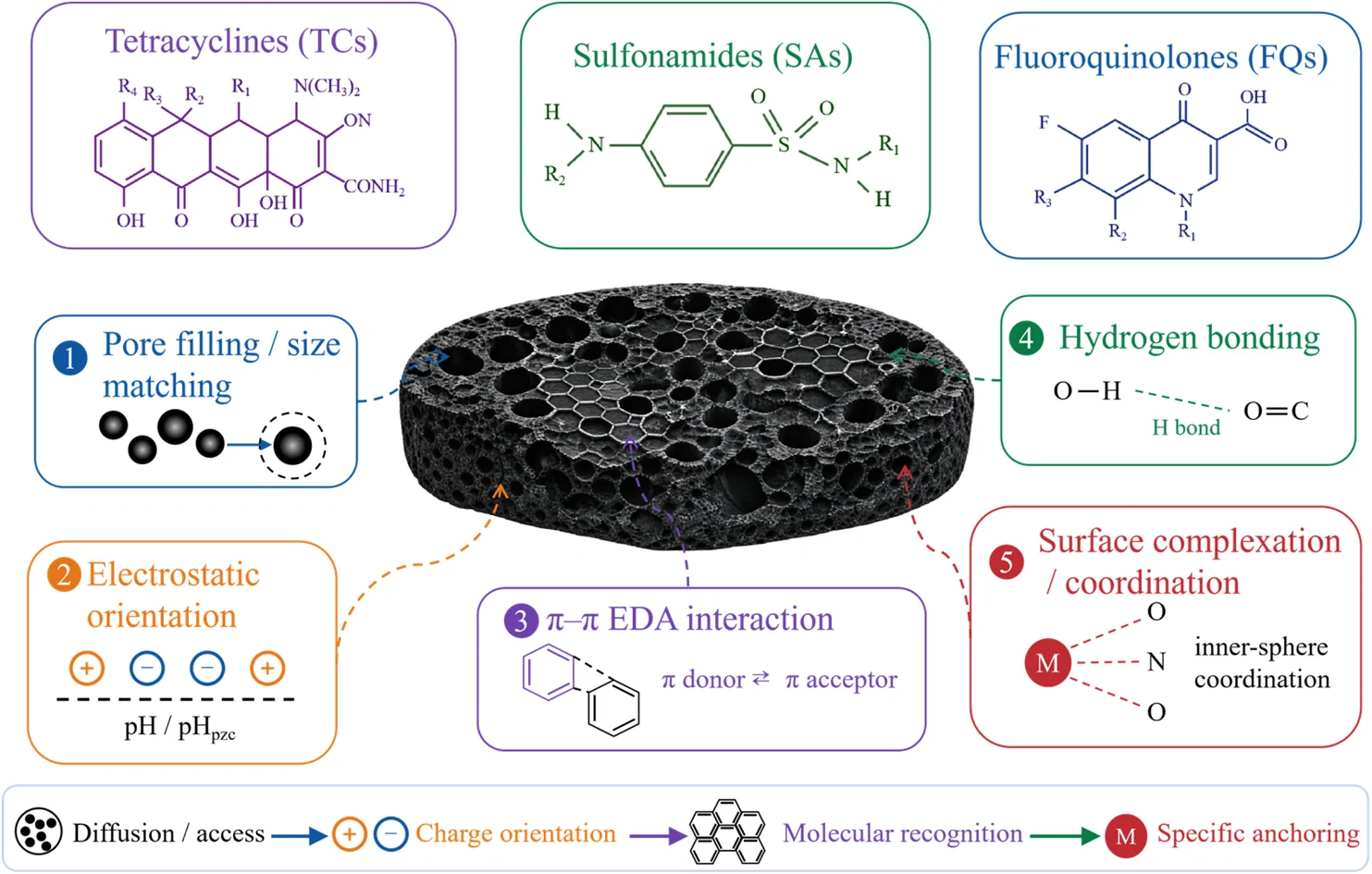

## 1. Introduction

Antibiotics are extensively used in human medicine, livestock production, aquaculture, and pharmaceutical manufacturing. However, their incomplete metabolism, continuous discharge, and insufficient removal in conventional wastewater treatment systems have resulted in their widespread occurrence in aquatic environments.^1–4^ Antibiotic residues have been detected in municipal wastewater, hospital effluents, livestock wastewater, aquaculture water, surface water, groundwater, sediments, and drinking-water-related environments, indicating that water systems act as important reservoirs and transport pathways for these contaminants.^1,2,5^ More importantly, the environmental risk of antibiotics is not limited to their direct toxicity. Long-term exposure to trace antibiotics may disturb microbial communities and promote the spread of antibiotic-resistant bacteria and antibiotic resistance genes, which has become a growing One Health concern.^3,4,6^

Among various antibiotic classes, tetracyclines (TCs), sulfonamides (SAs), and fluoroquinolones (FQs) are particularly representative because they are widely used, frequently detected, structurally diverse, and ionizable under environmentally relevant pH conditions.^2,5,7^ Their different molecular sizes, aromatic structures, functional groups, hydrophobicity, and dissociation constants also make them suitable model contaminants for comparing class-dependent adsorption behavior.^7–9^

Biological treatment, membrane filtration, advanced oxidation, photocatalysis, electrochemical processes, and adsorption have all been investigated for antibiotic removal.^2,10–12^ Among these approaches, adsorption is particularly attractive for micropollutant control because of its operational simplicity, relatively low energy demand, broad applicability, and compatibility with existing treatment processes.^5,13^ Biochar is a promising adsorbent platform because it combines a porous carbon structure and tunable surface chemistry with abundant waste-derived feedstocks and potential benefits for waste valorization and resource recovery.^14–17^

Pristine biochar, however, often exhibits variable adsorption performance because its pore structure, surface functionality, charge characteristics, and regeneration behavior depend strongly on feedstock type and pyrolysis conditions.^16–19^ Modification has therefore become an important strategy for tailoring pore architecture, surface chemistry, electronic properties, coordination sites, and adsorbent recoverability. Current approaches include alkali activation, acid and salt activation, metal and magnetic compositing, heteroatom doping, and hybrid functionalization.^20–30^ Nevertheless, an increase in adsorption capacity does not necessarily translate into practical applicability, because modification may also increase chemical consumption, synthesis complexity, regeneration difficulty, modifier or metal leaching, and life-cycle burden.^15,19^

Recent reviews have substantially advanced the understanding of biochar-based antibiotic remediation from complementary perspectives, including antibiotics and antibiotic resistance genes,^4,6^ functionalized biochars,^7^ class-resolved removal mechanisms and engineering applications,^31^ magnetic biochars for antibiotic adsorption and catalytic removal,^25^ broader preparation–modification–application frameworks for biochar-derived adsorbents,^32^ engineered biochar and composite systems encompassing adsorption, degradation, and ARG-related issues,^33^ and class-specific assessments of tetracyclines and sulfonamides.^8,34,35^ The scope and analytical emphasis of the most relevant recent reviews are systematically compared in Table S1. These contributions provide an important foundation for the field; however, their differences in material scope, pollutant scope, and treatment pathways make it difficult to extract directly comparable design principles for modified biochars used specifically as adsorbents. In particular, class-resolved differences among TCs, SAs, and FQs remain insufficiently synthesized within a common quantitative framework that simultaneously considers modification strategy, pH-dependent molecular speciation, adsorption-capacity distributions, interfacial interactions, and practical treatment constraints. Moreover, reported maximum adsorption capacities are often compared across studies despite substantial differences in initial antibiotic concentration, adsorbent dosage, pH, contact time, temperature, ionic strength, and model-fitting conditions. A focused comparative framework is therefore needed that moves beyond cataloguing high-capacity materials and instead connects antibiotic molecular properties, biochar modification, adsorption behavior, and engineering applicability.

Accordingly, this review focuses specifically on modified and engineered biochars for the adsorption of TCs, SAs, and FQs and provides four complementary levels of analysis. First, adsorption performance is compared at the antibiotic–adsorbent-system level, with reported capacities interpreted as performance distributions rather than absolute rankings across heterogeneous experimental conditions. Second, five major modification strategies—alkali activation, acid and salt activation, metal and magnetic compositing, heteroatom doping, and hybrid advanced compositing—are compared across the three antibiotic classes to identify class-dependent structure–performance relationships. Third, commonly reported interactions are integrated into a progressive interfacial framework in which pore accessibility enables retention, electrostatic interactions regulate molecular approach and orientation, π–π electron donor–acceptor interactions and hydrogen bonding provide molecular recognition and stabilization, and surface coordination contributes site-specific anchoring. Finally, adsorption performance is distinguished from application readiness by considering real-water matrix effects, regeneration stability, leaching risk, adsorbent recovery, continuous-flow operation, preparation complexity, techno-economic feasibility, and life-cycle implications. Through this integrated antibiotic class–modification–interaction–performance–application framework, the review aims to provide more transferable design principles for developing modified biochars for antibiotic-contaminated water treatment. The quantitative evidence base of this review comprised 50 primary publications on modified biochars for the adsorption of TCs, SAs, and FQs. Adsorption capacities, optimal pH values, isotherm and kinetic models, and reported mechanisms were analyzed at the antibiotic–adsorbent-system level where appropriate. Detailed literature-search procedures, eligibility criteria, data extraction, and the SI search of recent review literature are provided in Section S1 of the SI.

## 2. Molecular framework of antibiotic adsorption on modified biochars

### 2.1. Progressive interfacial adsorption framework

**Fig. 1:**
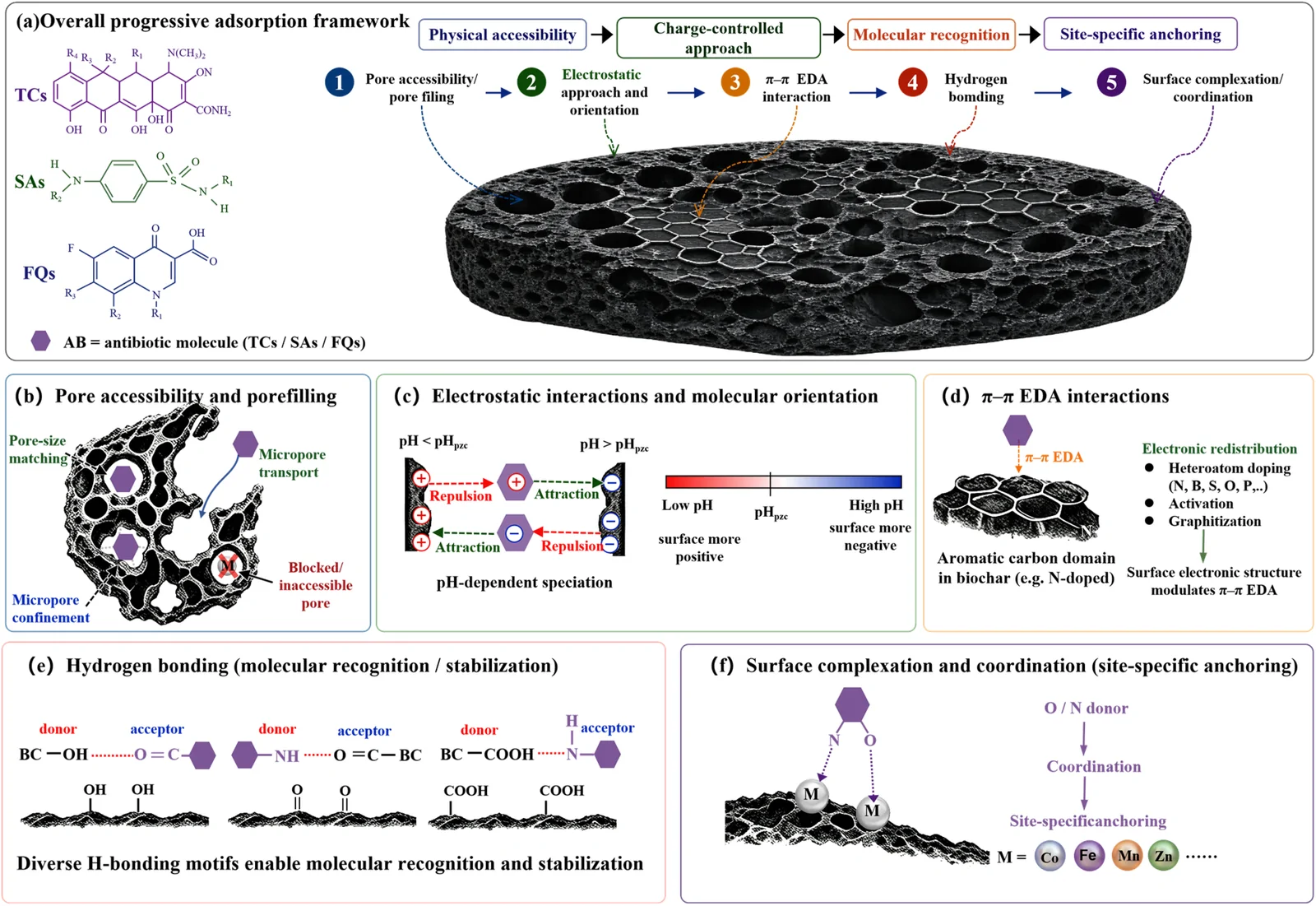
Progressive molecular framework for antibiotic adsorption on modified biochars: (a) overall progressive adsorption framework; (b) pore accessibility and pore filling; (c) electrostatic interactions and molecular orientation; (d) π–π electron donor–acceptor (EDA) interactions; (e) hydrogen bonding for molecular recognition and stabilization; and (f) surface complexation and coordination for site-specific anchoring.

Antibiotic adsorption on modified biochars is governed by coupled interfacial processes rather than by a single isolated interaction.^36,37^ As illustrated in Fig. 1, the adsorption process can be interpreted as a progressive framework in which pore accessibility first provides spatial domains for molecular retention, electrostatic interactions regulate molecular approach and orientation, π–π electron donor–acceptor (EDA) interactions and hydrogen bonding contribute to molecular recognition and stabilization, and surface complexation or coordination provides site-specific anchoring in selected metal-containing systems. Thus, adsorption of TCs, SAs, and FQs should be understood as the combined outcome of molecular accessibility, interfacial compatibility, and specific binding rather than as the consequence of a single dominant force.

**Fig. 2:**
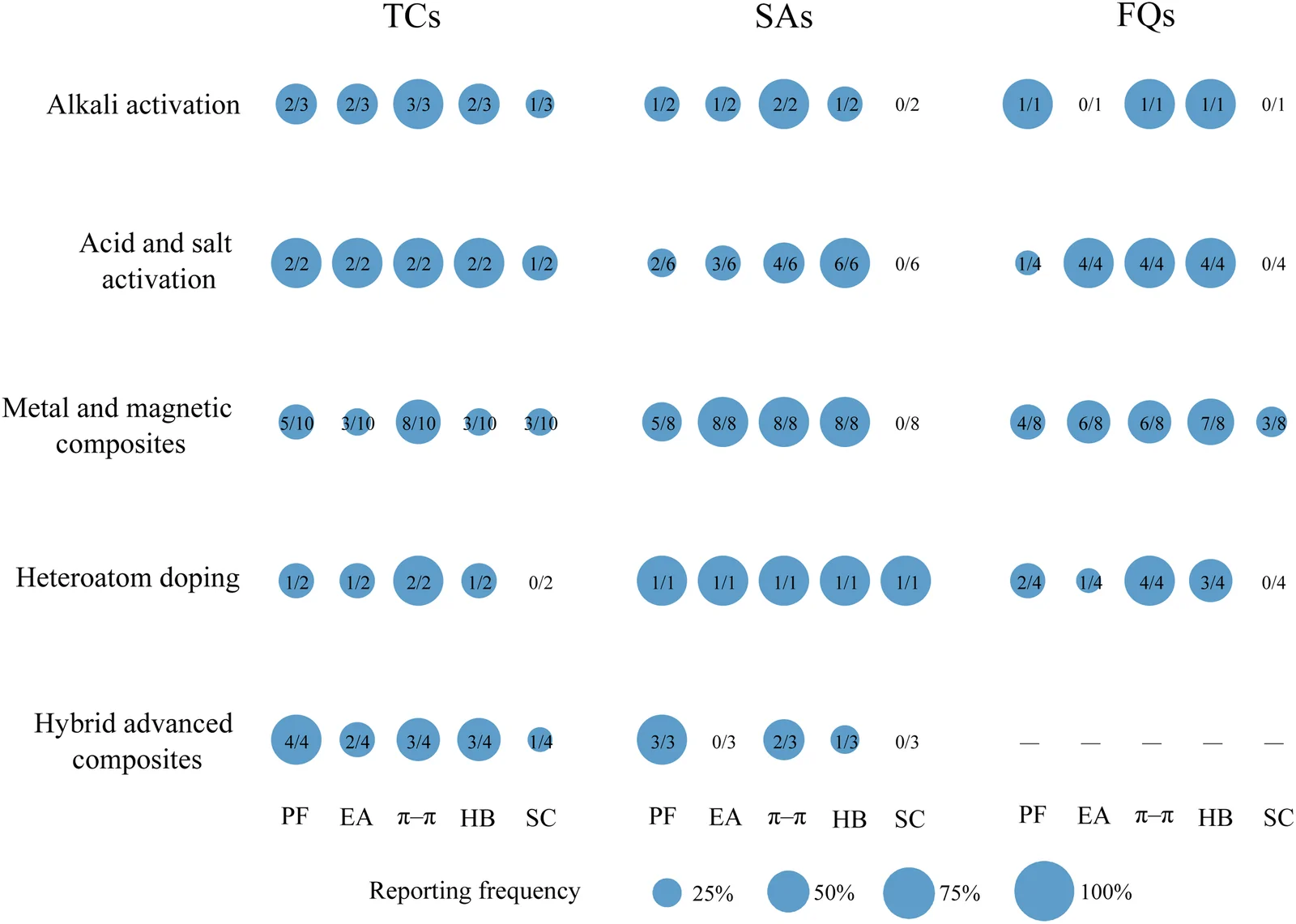
Mechanism-reporting matrix for antibiotic adsorption on modified biochars across modification strategies and antibiotic classes. Bubble area represents the percentage of antibiotic–adsorbent entries within each subgroup reporting a given mechanism, and labels indicate the number of entries reporting the mechanism/total number of entries in the subgroup. PF, pore filling; EA, electrostatic interaction; π–π, π–π electron donor–acceptor interaction; HB, hydrogen bonding; SC, surface complexation/coordination. Reporting frequency reflects the prevalence of mechanistic assignment in the compiled literature and should not be interpreted as energetic contribution. Dashes indicate no eligible entries.

The relative occurrence of these pathways also depends on both biochar modification strategy and antibiotic class. Fig. 2 summarizes the frequency with which pore filling (PF), electrostatic interaction (EA), π–π EDA interaction, hydrogen bonding (HB), and surface complexation/coordination (SC) were reported for the antibiotic–adsorbent entries compiled in this review. The matrix therefore complements the conceptual sequence in Fig. 1 by showing how mechanistic assignments vary across material–antibiotic combinations. Importantly, the reporting frequency of a mechanism should not be interpreted as its quantitative energetic contribution to adsorption.

### 2.2. Pore accessibility and pore filling

Pore filling is the spatial foundation of antibiotic adsorption. Before specific interactions such as π–π stacking or metal coordination occur, antibiotic molecules must first diffuse into accessible internal pores or reach external adsorption domains. Chemical activation, acid/salt activation, heteroatom doping, and hybrid modification commonly increase specific surface area, generate micro-/mesoporous channels, and expose additional adsorption sites.^6,13,14^

However, pore filling cannot be simplified as “higher BET surface area equals higher adsorption”. Micropores generate strong confinement potentials, but they may exclude bulky molecules if the pore entrance is too narrow. Mesopores facilitate mass transfer and are especially important for larger TCs and FQs.^13,17^ Macropores contribute less to adsorption energy but can reduce diffusion resistance. Thus, an optimized hierarchical pore structure is more meaningful than maximum surface area alone. Therefore, pore filling provides the physical storage basis, whereas surface chemistry determines whether the retained antibiotics are weakly partitioned or strongly stabilized.

### 2.3. Electrostatic interactions and molecular orientation

Electrostatic interactions mainly control the initial approach and orientation of antibiotic molecules at the biochar–water interface. This mechanism depends on solution pH, antibiotic dissociation constants, and the point of zero charge of modified biochars.^6,38^ TCs, SAs, and FQs are all ionizable, but their charge states differ significantly. TCs may exist as cationic, zwitterionic, or anionic species; SAs become increasingly anionic under neutral-to-alkaline conditions; and FQs can shift among cationic, zwitterionic, and anionic forms because of carboxyl and piperazinyl groups. Meanwhile, biochar surfaces may become positively or negatively charged depending on pH and surface functional groups.

Therefore, electrostatic attraction should be viewed as a “gating mechanism”: it determines whether antibiotics can approach and orient favorably on the surface, but it rarely dominates the final adsorption strength alone. Ionic strength, competing ions, and dissolved organic matter may further weaken electrostatic attraction by charge screening or site competition.^39^ The detailed class-dependent effects of pH and antibiotic speciation are discussed separately in Section 5.1; here, electrostatics are considered principally as the interfacial step controlling whether molecules can efficiently reach and orient toward subsequent recognition sites.

### 2.4. π–π Electron donor–acceptor interactions

π–π EDA interactions are particularly important mechanisms for aromatic antibiotic adsorption on carbon-based materials. Modified biochars contain condensed aromatic domains, graphitic fragments, and defect-rich carbon structures.^4,17^ TCs and FQs have extended conjugated or aromatic skeletons, while SAs contain aromatic rings with electron-withdrawing sulfonamide groups. When antibiotic aromatic rings approach biochar aromatic domains, π–π stacking can occur. More importantly, the interaction is often an electron donor–acceptor process rather than simple physical overlap. The electron density of the biochar surface and the electron-donating or electron-withdrawing substituents of antibiotic molecules jointly determine the strength of this interaction.

Therefore, π–π EDA interactions should be described as electronically regulated molecular recognition between antibiotic aromatic structures and engineered carbon surfaces.^40^ The relative importance of π–π interactions differs among antibiotic classes. TCs, with fused conjugated rings, can interact strongly with graphitized or highly aromatic biochars, especially when molecular planarity and pore accessibility are favorable. FQs contain quinolone cores and often show strong π–π affinity, but their adsorption is also strongly regulated by pH-dependent zwitterionic forms and polar functional groups. SAs generally have less extended aromatic systems, so π–π interaction may contribute but often works together with hydrogen bonding, electrostatic attraction, hydrophobic interaction, and pore confinement.^41,42^

Mechanistic assignment of π–π EDA interactions should still be cautious. In many studies, this mechanism is inferred from increased aromaticity, FTIR shifts of aromatic C=C bands, Raman *I*_D_/*I*_G_ changes, or high adsorption of aromatic antibiotics. These are useful but indirect indicators. Because π–π EDA may overlap with hydrophobic partitioning, pore filling, and hydrogen bonding, stronger evidence should include XPS analysis of electron-density changes, solid-state NMR, competitive adsorption using aromatic and non-aromatic probes, DFT calculations, molecular dynamics simulations, and charge-density difference analysis. Future studies should quantify π–π contributions rather than simply listing them as one of several possible mechanisms.

### 2.5. Hydrogen bonding

Hydrogen bonding provides an additional molecular recognition pathway because both antibiotics and modified biochars contain proton donors and acceptors. TCs contain hydroxyl, phenolic, carbonyl, amide, and amino groups; SAs contain amino and sulfonyl groups; and FQs contain carboxyl, carbonyl, and nitrogen-containing groups.^6,14^ Modified biochars can provide –OH, –COOH, C=O, –NH_2_, phosphate groups, and other O/N-containing moieties. Therefore, multiple hydrogen-bonding configurations can form at the solid–water interface.

However, hydrogen bonding is sensitive to hydration, pH, steric hindrance, and natural organic matter. It rarely acts as the sole driving force; instead, it stabilizes adsorbed complexes and reinforces molecular recognition after pore filling and electrostatic approach.^43,44^ Within the progressive framework in Fig. 1, hydrogen bonding is therefore interpreted primarily as a molecular-recognition and stabilization pathway rather than as an isolated adsorption mechanism.

### 2.6. Surface complexation and coordination

Surface complexation and coordination can be regarded as key mechanisms distinguishing metal/magnetic modified biochars from ordinary carbonaceous biochars. When Fe, Co, Mn, Zn, La, MgFe_2_O_4_, Fe_3_O_4_, ferrites, or bimetallic oxides are introduced, the biochar surface changes from a mainly carbon-based adsorption platform to a coordination-active interface.^14,45,46^ Metal centers can serve as Lewis acidic sites and bind electron-donating groups in antibiotics, including carbonyl, hydroxyl, carboxylate, amino, keto–enol, sulfonamide oxygen, and heterocyclic nitrogen groups. This mechanism is particularly important for TCs and FQs because they possess multiple O/N donor atoms capable of chelation or inner-sphere coordination.

Metal incorporation may also modify local surface charge, acid–base properties, and interfacial hydration, thereby influencing electrostatic interactions and hydrogen bonding in parallel with direct coordination.^45,46^ Therefore, metal-containing biochars should not automatically be interpreted as purely coordination-controlled adsorbents; their performance may still depend strongly on pore accessibility and the surrounding carbon surface.

Nevertheless, surface complexation is sometimes over-assigned. The presence of metals, enhanced adsorption after metal loading, or pH-dependent behavior alone cannot prove inner-sphere coordination. More reliable evidence should include XPS shifts of Fe 2p, Co 2p, Mn 2p, Zn 2p, O 1s, or N 1s peaks; FTIR changes in carbonyl, carboxyl, hydroxyl, or amino bands; metal-site blocking experiments; EXAFS analysis; and DFT-supported binding geometries.^17,45^ Without such evidence, surface complexation should be described cautiously as a possible or contributing mechanism.

Overall, antibiotic adsorption on modified biochars follows a clear mechanistic sequence: pores provide access and storage, electrostatics regulates approach, π–π EDA interactions and hydrogen bonding enable molecular recognition, and metal coordination confers specificity and strong anchoring. This mechanistic sequence represents a central conceptual contribution of this review. Fig. 2 complements this conceptual sequence by showing that the reported occurrence of individual mechanisms varies across modification strategies and antibiotic classes. Accordingly, mechanistic assignments throughout this review should be treated as evidence-weighted interpretations rather than as a checklist in which every reported interaction contributes equally.

## 3. Biochar modification strategies

**Fig. 3:**
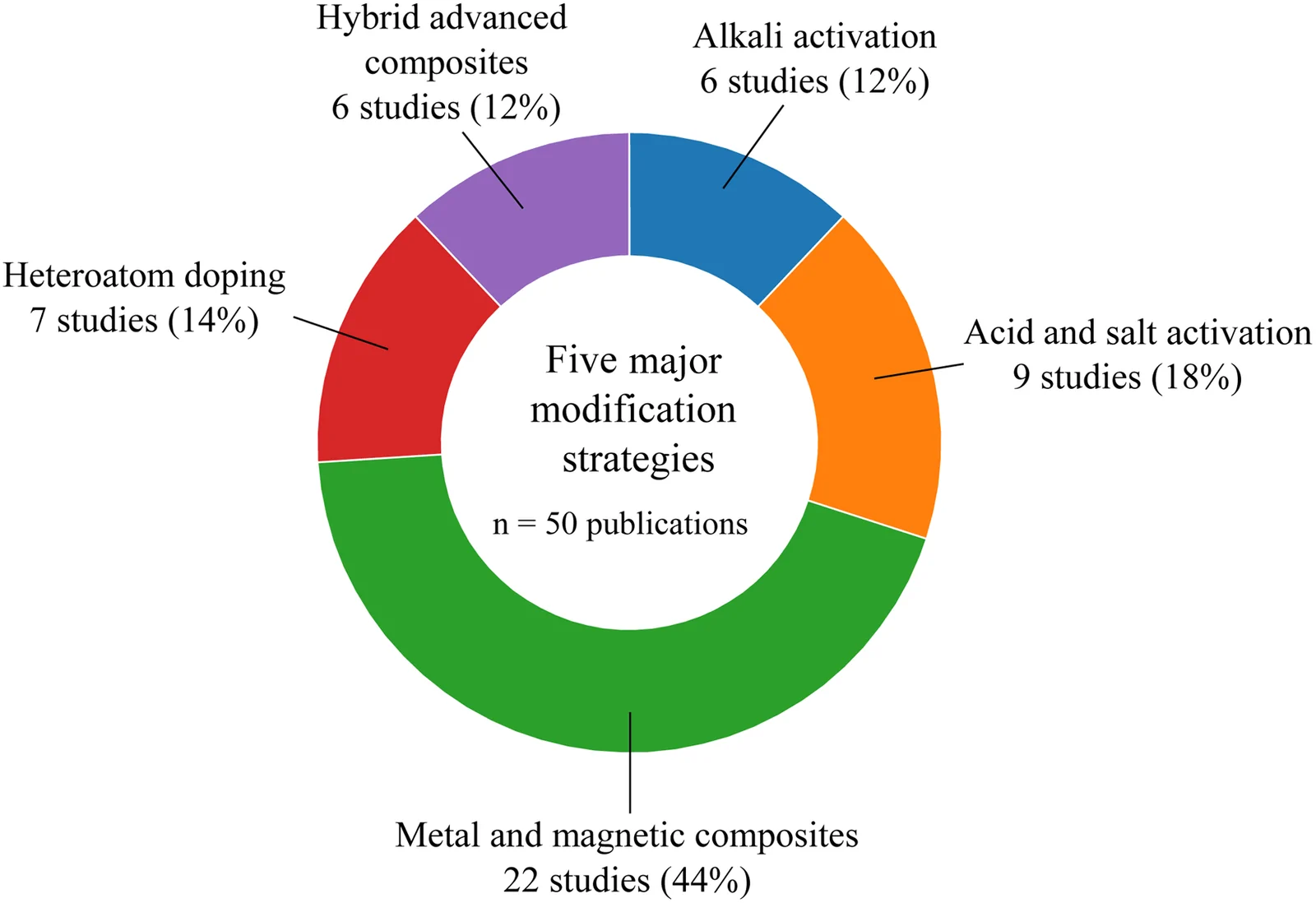
Distribution of modification strategies among the collected modified biochar publications for antibiotic adsorption.

As summarized in Fig. 3, the 50 retained publications in this review can be classified into five modification strategies. Metal and magnetic composites are the dominant category, accounting for 22 publications (44%), followed by acid and salt activation with 9 publications (18%), heteroatom doping with 7 publications (14%), alkali activation with 6 studies (12%), and hybrid advanced composites with 6 studies (12%). This distribution indicates that recent biochar design for antibiotic adsorption has moved beyond simple surface-area enhancement toward the coordinated regulation of pore architecture, surface charge, electronic structure, functional groups, and recoverability. Table 1 further shows that high adsorption capacity is usually achieved when structural accessibility and chemically specific interfacial sites are optimized simultaneously.

**Table 1:** Representative modified biochars for the adsorption of typical antibiotics and their key characteristics^a^

Feedstock	Modification strategy	Representative modifier	Target antibiotic	Pyrolysis/preparation condition	BET surface area (m^2^ g^−1^)	Opt. pH	*Q*_max_ (mg g^−1^)	Dominant mechanism	Ref.
*Chlorella vulgaris*	Alkali activation	Na_2_HPO_4_ + KOH	Tetracycline hydrochloride	Na_2_HPO_4_ impregnation; KOH/biomass = 0.75 : 1; 700 °C, 2 h, N_2_	1873.78	3	662.91	π–π	21
*Enteromorpha prolifera*	Alkali activation	KOH	Sulfamethoxazole	Pre-pyrolysis 500 °C, 5 h; biochar/KOH = 1 : 0.5; 800 °C, 2 h, N_2_	2172.08	5	744.32	PF, EA, HB, π–π	49
Highland barley straw	Acid and salt activation	ZnCl_2_	Tetracycline hydrochloride	2 M ZnCl_2_; 750 °C, 2 h, N_2_	862.85	3	531.40	PF, SC, EA, HB, π–π	99
Wheat straw	Acid and salt activation	ZnCl_2_	Sulfamethoxazole	ZnCl_2_/wheat straw = 1 : 1; 800 °C, 4 h, N_2_	1328.00	4	597.57	PF, EA, HB, π–π	42
Cotton husk	Acid and salt activation	H_3_PO_4_	Ciprofloxacin	30% H_3_PO_4_ impregnation; 600 °C, 120 min, N_2_	316.10	6	572.80	PF, EA, HB, π–π	52
*Enteromorpha prolifera*	Metal and magnetic composites	Co(NO_3_)_2_	Tetracycline hydrochloride	Co impregnation at 80 °C, 3 h; 900 °C, 2 h, N_2_	NR	5	940.40	SC, EA, π–π	74
*Enteromorpha prolifera*	Metal and magnetic composites	KOH + FeSO_4_/FeCl_3_	Sulfamethoxazole	Pre-pyrolysis 500 °C, 5 h; co-pyrolysis 800 °C, 2 h; Fe^2+^/Fe^3+^ coprecipitation	1512.53	7	502.20	PF, EA, HB, π–π	23
Delignified bamboo	Metal and magnetic composites	Fe_3_O_4_ + KOH	Norfloxacin	Hydrothermal magnetization; 400 °C, 2 h; KOH activation at 800 °C, 2 h	1253.66	3	458.43	EA, HB, π–π	24
Furfural residue lignin	Heteroatom doping	KOH + melamine	Tetracycline	KOH/melamine activation; 800 °C, 1 h, N_2_	3463.87	4	2242.00	PF, EA, HB, π–π	26
Longan seed	Heteroatom doping	H_3_BO_3_ + KOH	Lomefloxacin	H_3_BO_3_ pre-carbonization at 600 °C; KOH activation at 850 °C, 1 h, N_2_	2657.30	7	1295.40	π–π	63
Corn straw residue	Hybrid advanced composites	Microbial + *p*-TsOH pre-treatment	Tetracycline/sulfadiazine	500 °C carbonization; KOH/NaOH activation; 800 °C, N_2_	2693/2672	5/acidic	1216/1083	PF	29
Natural loofah cellulose	Hybrid advanced composites	Polydopamine + ZIF-67	Tetracycline	PDA coating; ZIF-67 *in situ* growth; 650 °C, 2 h, N_2_	444.59	7	662.25	PF, EA, HB, π–π	30

a PF, pore filling; SC, surface complexation (including metal/functional group complexation and inner-sphere coordination); EA, electrostatic attraction/interaction; HB, hydrogen bonding; π–π, π–π interactions (including π–π electron donor–acceptor interactions). For concise presentation in the main text, isotherm models, kinetic models, and regeneration data are summarized in Table S2 rather than repeated here. The selected entries were condensed from the compiled dataset in Table S2.

To avoid repeating the molecular mechanisms defined in Section 2, the five strategies below are discussed primarily in terms of the physicochemical properties they create, while their corresponding interaction pathways are interpreted through Fig. 1 and 2.

### 3.1. Alkali activation

Alkali activation, commonly using KOH or NaOH, mainly reconstructs the carbon skeleton through chemical etching, gasification reactions, and removal of unstable organic fractions. This process greatly increases specific surface area, creates abundant micro-/mesopores, exposes aromatic carbon domains, and generates defect-rich surfaces.^47,48^ In Table 1, Na_2_HPO_4_/KOH-activated *Chlorella vulgaris* biochar and KOH-activated *Enteromorpha prolifera* biochar both show high BET surface areas and high adsorption capacities, illustrating the strong pore-development capability of alkali activation.^21,49^ However, excessive alkali dosage may collapse pore walls or remove oxygen-containing groups, so alkali activation should be optimized to balance porosity and surface chemistry.^50^ Accordingly, the principal design objective of alkali activation is to create accessible hierarchical porosity while preserving chemically useful surface domains; the associated adsorption interactions are discussed in Section 2.

### 3.2. Acid and salt activation

Acid and salt activation modifies biochar through dehydration, crosslinking, aromatization, mineral templating, and volatile-release regulation.^20,22^ H_3_PO_4_ usually promotes dehydration and introduces P-/O-containing functional groups, whereas ZnCl_2_ facilitates micropore–mesopore formation by suppressing tar generation and acting as a pore-forming template.^22,51^ These changes increase not only pore volume and surface area but also the density of polar sites such as –OH, –COOH, P–O, and C=O groups. The ZnCl_2_-activated wheat-straw biochar and H_3_PO_4_-modified cotton-husk biochar in Table 1 illustrate that acid/salt activation can simultaneously improve pore structure and surface functionality toward sulfamethoxazole and ciprofloxacin.^42,52^ Therefore, this strategy should be described as a pore-chemistry co-regulation route rather than simple physical activation.^53,54^ Its adsorption significance is consequently determined by the combined changes in accessibility, polarity, and surface functionality rather than by a single predefined interaction mechanism.

### 3.3. Metal and magnetic composites

Metal and magnetic modification is the most frequently reported strategy in the present dataset. Loading Fe_3_O_4_, ferrites, Zn/Fe oxides, Co species, Mn oxides, or bimetallic phases changes biochar from a mainly carbonaceous adsorbent into a coordination-active and magnetically recoverable composite.^55^ Structurally, metal oxides may occupy or create pores depending on loading amount and preparation route.^23,56,57^ Chemically, they introduce Lewis acidic metal centers, metal-OH groups, variable surface charge, and inner-sphere complexation sites.^24,58^ Representative systems in Table 1, including Fe-modified algae biochar, Fe_3_O_4_/KOH-modified bamboo biochar, and Fe/Zn-functionalized biochar, show that metal/magnetic modification can combine carbonaceous pore domains, chemically differentiated metal-containing interfaces, and magnetic separation.^23,24,59^ The presence of a metal phase, however, should not by itself be taken as proof that coordination dominates adsorption; the evidence requirements for assigning surface complexation are discussed in Section 2.6. Nevertheless, this strategy should also be discussed with caution because metal leaching, active-site blockage, and long-term regeneration stability may affect practical application.^60–62^

### 3.4. Heteroatom doping

Heteroatom doping regulates biochar primarily at the electronic and surface-chemical levels. N, B, P, S, or F atoms can be incorporated into the carbon lattice or anchored as surface functional groups, thereby altering charge density, defect density, polarity, acid–base properties, and π-electron distribution. N-doping can introduce pyridinic-N, pyrrolic-N, and graphitic-N sites,^26,27^ while B-, F-, P-, and S-containing structures create distinct electronic and chemical environments at the carbon surface. In Table 1, the ultrahigh tetracycline capacity of KOH/melamine-modified lignin biochar and the high fluoroquinolone uptake of B-assisted longan-seed biochar suggest that heteroatom doping is especially useful when adsorption depends on electronic matching rather than surface area alone.^26,28,63^ Accordingly, heteroatom doping is best viewed as an electronic-structure and surface-polarity engineering strategy,^64,65^ with its adsorption consequences interpreted through the molecular-recognition framework in Section 2.

### 3.5. Hybrid advanced composites

Hybrid advanced composites integrate biochar with polymers, biomass pretreatments, ionic-liquid-derived species, MOF-related structures, or bio-based functional coatings.^66^ Compared with single activation or single doping, hybridization can simultaneously tune hierarchical porosity, surface functionality, interfacial stability, and pollutant selectivity.^29^ For example, microbial/*p*-TsOH pretreatment can improve biomass deconstruction and facilitate highly porous carbon formation, while polydopamine, chitosan, or MOF-derived components introduce abundant –NH_2_, –OH, metal-N, and metal-O sites.^29,30^ These multifunctional interfaces provide several types of adsorption domains within the same material;^67^ their respective interaction pathways are summarized in Fig. 1 and 2 rather than repeated here. The *p*-TsOH-pretreated corn-straw biochar and PDA/ZIF-67-derived loofah-cellulose biochar in Table 1 are typical examples of this multiscale interfacial engineering. However, hybrid composites often require multi-step synthesis and may involve higher reagent consumption, possible modifier release, and more complex regeneration. Thus, their advantages should be evaluated not only by *Q*_max_ but also by matrix tolerance, recyclability, leaching risk, and scalability.^68,69^

Overall, the modification strategies summarized in Fig. 3 reveal a clear design logic: alkali and acid/salt activation mainly optimize pore structure and accessible surface; metal/magnetic modification introduces coordination sites and recoverability; heteroatom doping tunes electronic structure and surface polarity; and hybrid composites integrate multiple interfaces into one adsorbent. These property-level differences provide the material basis for the class-resolved adsorption-performance comparison in Section 4, while their mechanistic implications are interpreted using the framework established in Section 2.

## 4. Class-resolved adsorption performance

**Fig. 4:**
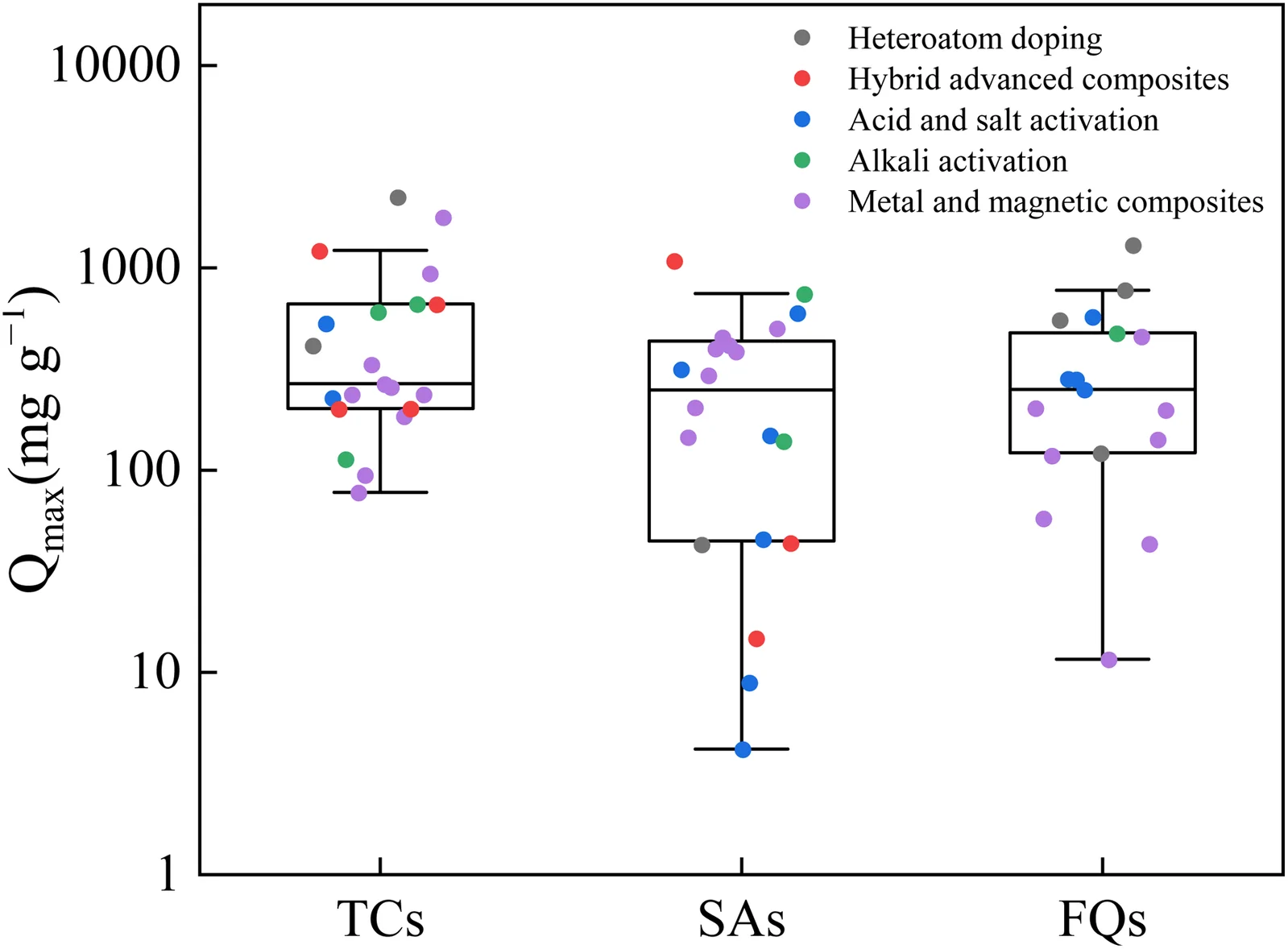
Distribution of maximum adsorption capacities of modified biochars for tetracyclines, sulfonamides, and fluoroquinolones. The *y*-axis is plotted on a logarithmic scale.

As shown in Fig. 4 and summarized in Table 2, modified biochars exhibited clear class-dependent adsorption performance toward typical antibiotics. The box-and-scatter plot provides a distribution-based comparison by visualizing not only the highest reported *Q*_max_ values but also the median, dispersion, and outliers of each antibiotic class. In the compiled dataset, *Q*_max_ ranged from 77.60 to 2242.00 mg g^−1^ for TCs, from 4.18 to 1083.00 mg g^−1^ for SAs, and from 11.62 to 1295.40 mg g^−1^ for FQs. Their medians were relatively close, approximately 266.94 mg g^−1^ for TCs, 249.34 mg g^−1^ for SAs, and 250.46 mg g^−1^ for FQs. However, the upper quartile and extreme values of TCs were higher, indicating that TCs are more likely to reach exceptionally high capacities after suitable modification. SAs showed the widest relative spread, with several low-capacity cases and a few high outliers, suggesting stronger dependence on material design and solution chemistry. Therefore, *Q*_max_ is better interpreted as a distribution rather than as isolated record values.^70–72^ In this section, the three antibiotic classes are compared primarily through their capacity distributions, high- and low-performance regimes, and sensitivity to material design, while the molecular interactions responsible for adsorption are interpreted through the framework established in Section 2.

**Table 2:** Comparative summary of adsorption performance, efficient modification strategies, dominant mechanisms, and interpretation cautions for typical antibiotic classes^a^

Antibiotic class	Representative molecules	*Q*_max_ range (mg g^−1^)	Common high-efficiency modification strategies	Main dominant mechanisms	Main interpretation caution
Tetracyclines (TCs)	Tetracycline; tetracycline hydrochloride	77.60–2242.00	Heteroatom doping; metal and magnetic composites; hybrid advanced composites; alkali activation	PF; π–π interaction; EA; HB; SC frequently involved	High upper-range capacities are often associated with strong multipoint interactions, but they may also depend on optimized batch conditions, high initial concentrations, and highly engineered materials. Direct ranking across studies should therefore be avoided
Sulfonamides (SAs)	Sulfamethoxazole; sulfadiazine; sulfamethazine; sulfamerazine	4.18–1083.00	Hybrid advanced composites; alkali activation; acid and salt activation; Fe-based metal/magnetic composites	PF; EA; HB; π–π interaction; SC in selected composite systems	Adsorption is highly sensitive to pH-regulated speciation, surface charge, and pore compatibility. Low-capacity cases indicate that functionalization alone may be insufficient without accessible pores and charge-compatible adsorption sites
Fluoroquinolones (FQs)	Ciprofloxacin; norfloxacin; levofloxacin; lomefloxacin; moxifloxacin; ofloxacin; enrofloxacin	11.62–1295.40	Heteroatom doping; acid and salt activation; alkali activation; metal and magnetic composites	π–π interaction; HB; EA; PF commonly involved; SC/IX in some metal-containing systems	Adsorption is strongly molecule-specific and controlled by amphoteric speciation, pore-size matching, surface polarity, and electronic structure. Magnetic recovery does not necessarily indicate high adsorption capacity

a PF, pore filling; SC, surface complexation, including metal/functional group complexation and inner-sphere coordination; EA, electrostatic attraction/interaction; HB, hydrogen bonding; IX, ion exchange; π–π, π–π interactions, including π–π electron donor–acceptor interactions. *Q*_max_ values should be interpreted as performance envelopes rather than absolute rankings because adsorption conditions, concentration ranges, pH, adsorbent dosages, and model fitting methods vary among studies.

### 4.1. Tetracyclines (TCs)

TCs showed the highest upper-capacity distribution in Fig. 4.

The maximum *Q*_max_ was 2242.00 mg g^−1^ for KOH/melamine-modified furfural-residue lignin biochar,^26^ followed by other high-capacity systems, including Mo/Fe-modified rice-straw biochar (1781.81 mg g^−1^),^73^ microbial/*p*-toluenesulfonic-acid-pretreated corn-straw-residue biochar, Co-modified *Enteromorpha prolifera* biochar, Na_2_HPO_4_/KOH-activated *Chlorella vulgaris* biochar, and PDA/ZIF-67-derived loofah-cellulose biochar.^21,29,30,74^ These examples indicate that high TC adsorption usually requires either strong pore development or multifunctional surface engineering. However, the boxplot also shows that TC adsorption is not uniformly high, because some Fe- or Fe/Zn-modified biochars remained below 100 mg g^−1^. Thus, metal loading or magnetic modification does not automatically enhance TC uptake if active sites are blocked or pore accessibility is poor.

The relatively strong adsorption of TCs is closely related to their molecular architecture. Tetracycline molecules contain a fused polycyclic skeleton and multiple hydroxyl, carbonyl, amide, phenolic, and dimethylamino groups.^8,75^ Rather than repeating the individual interaction pathways described in Section 2, these structural features are interpreted here as providing multiple points of compatibility with porous, aromatic, polar, and metal-containing adsorption domains. This multidentate binding ability explains why TCs show a stronger upper tail than SAs and FQs. Therefore, the key design principle for TCs is the coupled optimization of hierarchical porosity, aromatic carbon domains, and polar or metallic binding sites.^76,77^ The exceptionally high capacities of selected TC systems are therefore better viewed as the outcome of multifunctional interfacial compatibility than as evidence for any single dominant mechanism.

### 4.2. Sulfonamides (SAs)

Compared with TCs, SAs displayed lower and more scattered adsorption capacities. Their *Q*_max_ values ranged from 4.18 to 1083.00 mg g^−1^, and the lower tail was more obvious. Phosphogypsum-modified wood-chip biochar, chitosan/glutaraldehyde-modified coffee-ground biochar, biosurfactant-modified sludge biochar, and some low-porosity functionalized materials showed weak SA uptake.^67,78,79^ These cases indicate that adding functional groups alone is insufficient when the carbon matrix lacks pore volume, accessible aromatic domains, or charge-compatible sites. In this sense, SAs are a useful probe for judging whether modification truly improves the adsorption interface.

The moderate adsorption of many SAs is associated with their smaller size, higher polarity, and pH-sensitive ionization. Sulfonamides contain an aniline moiety and a sulfonamide group, but they lack the multiple chelating O/N sites present in TCs and many FQs. The wider dispersion of SA capacities therefore reflects a stronger dependence on molecular ionization, surface-charge compatibility, and pore accessibility, rather than a single adsorption mechanism.^6,80^ The detailed pH-dependent speciation of SAs and its effect on surface-charge matching are discussed in Section 5.1 rather than repeated here. The high outliers in Fig. 4, such as pretreated corn-straw-residue biochar and KOH-activated *Enteromorpha* biochar, show that SAs can be efficiently removed when hierarchical pores and surface affinity are simultaneously optimized.^41,42,49,81^ Thus, the defining feature of SAs in the present dataset is not simply lower adsorption capacity, but greater sensitivity to material design and experimental conditions.

### 4.3. Fluoroquinolones (FQs)

FQs showed an intermediate but highly material-dependent capacity distribution. Their *Q*_max_ values ranged from 11.62 to 1295.40 mg g^−1^, and their median was close to those of TCs and SAs. However, the distribution contained both very low magnetic-biochar values and high heteroatom-doped or acid-activated values. The highest FQ capacity was obtained for H_3_BO_3_/KOH-modified longan-seed biochar toward lomefloxacin.^63^ Other effective systems included self-N-doped waste-peanut-meal biochar, N–F co-doped apple-branch biochar, H_3_PO_4_-functionalized cotton-husk biochar, and ZnCl_2_-activated bamboo biochar.^27,28,52,82^ These results indicate that FQs respond well to modifications that create electron-regulated aromatic domains and polar binding sites. More generally, the high- and low-capacity extremes suggest that FQ adsorption is especially sensitive to how effectively a modification preserves accessible pores while tuning surface polarity and electronic structure.

The variability of FQ adsorption originates from their amphoteric and aromatic structures. FQs contain a quinolone ring, carboxyl and carbonyl groups, and often piperazinyl or other nitrogen-containing groups.^9^ The molecular interaction pathways arising from these features are discussed in Section 2; in the present performance comparison, their main implication is the strong dependence of FQ uptake on pore-size compatibility, surface chemistry, and pH-regulated speciation. However, some Fe_3_O_4_-containing biochars offered magnetic recovery but limited *Q*_max_, suggesting that recoverability and adsorption strength are not always positively correlated.^28,83^ Thus, FQ-oriented design should prioritize pore-size compatibility, surface polarity, and pH-dependent speciation rather than simply increasing metal loading or magnetism.^84^

### 4.4. Comparative analysis across systems

Across the three antibiotic classes, heteroatom-doped and strongly activated biochars showed the most evident overall enhancement, although the smaller number of heteroatom-doped samples should be acknowledged. Heteroatom doping produced the highest median *Q*_max_ among modification strategies and generated the strongest outliers for TCs and FQs. Its advantage is interpreted here as the combined result of electronic-structure and surface-chemical regulation described in Section 3, rather than as evidence for any single interaction pathway. Alkali activation was also consistently effective because it directly created high surface area, micro-/mesopores, and exposed aromatic domains. Hybrid advanced composites produced very high capacities in selected cases, but their distribution was broad, meaning that hybridization is beneficial only when it improves real interfacial accessibility instead of adding inactive mass. Metal and magnetic composites were the most frequently studied and offered separation advantages, but their median capacity was not the highest; excessive metal loading may block pores, reduce accessible carbon surface, or introduce leaching risks. These results reinforce the distinction established in Section 3 between modification frequency, material functionality, and adsorption-capacity performance.

Among antibiotic classes, TCs appear more readily adsorbed by suitably engineered biochars under the compiled experimental conditions. Although the medians of the three classes were similar, TCs had the highest mean, upper quartile, and maximum *Q*_max_, reflecting their stronger tendency to form multipoint interactions with engineered carbon surfaces.^85^ FQs rank next: they can reach high capacities when surface electronic structure and pH conditions are favorable, but their adsorption is more molecule-specific.^86^ SAs are the most variable and are more strongly constrained by pH, surface charge, and pore compatibility.^6,80^ Nevertheless, the compiled distributions should not be reduced to a universal ranking of TCs > FQs > SAs. Instead, antibiotic class primarily influences the range of material and solution conditions under which high-capacity adsorption can be achieved.

A high *Q*_max_, however, does not necessarily mean better engineering applicability. *Q*_max_ is usually derived from batch isotherms under optimized single-solute conditions, often using high initial concentrations, controlled pH, long contact times, and ultrapure water. These conditions may overestimate performance in real wastewater, where dissolved organic matter, inorganic ions, suspended solids, and coexisting pollutants compete for sites or block pores.^87^ High-capacity materials may also require strong activators, high pyrolysis temperatures, multi-step synthesis, intensive washing, or expensive modifiers, which can reduce sustainability and increase secondary-pollution risks.^88^ Therefore, *Q*_max_ should be evaluated together with adsorption rate, working capacity at environmentally relevant concentrations, regeneration efficiency, column breakthrough behavior, matrix tolerance, leaching, adsorbent loss, and preparation cost.^88,89^

Cross-study comparison is further affected by methodological inconsistency. Different studies used different adsorbent dosages, initial concentrations, pH values, ionic strengths, temperatures, particle sizes, equilibrium times, and isotherm models. Because Langmuir-derived *Q*_max_ is sensitive to concentration range and model selection, values from different papers should be compared as capacity envelopes rather than strict material rankings. The most reliable comparisons are paired tests within the same study. Therefore, Fig. 4 provides a useful overview of performance distribution, but its interpretation should emphasize trends rather than absolute superiority. Overall, future high-performance biochars should move from “maximum *Q*_max_ pursuit” toward antibiotic-class-specific and application-oriented design: TCs require multipoint functional binding, SAs require charge-compatible pore-surface matching, and FQs require electronic-structure tuning plus pH-adapted interfacial sites. Together with Sections 2 and 3, the present analysis links antibiotic class, material modification, interfacial compatibility, and adsorption performance within a single comparative framework.

## 5. Environmental and adsorption-process factors

**Fig. 5:**
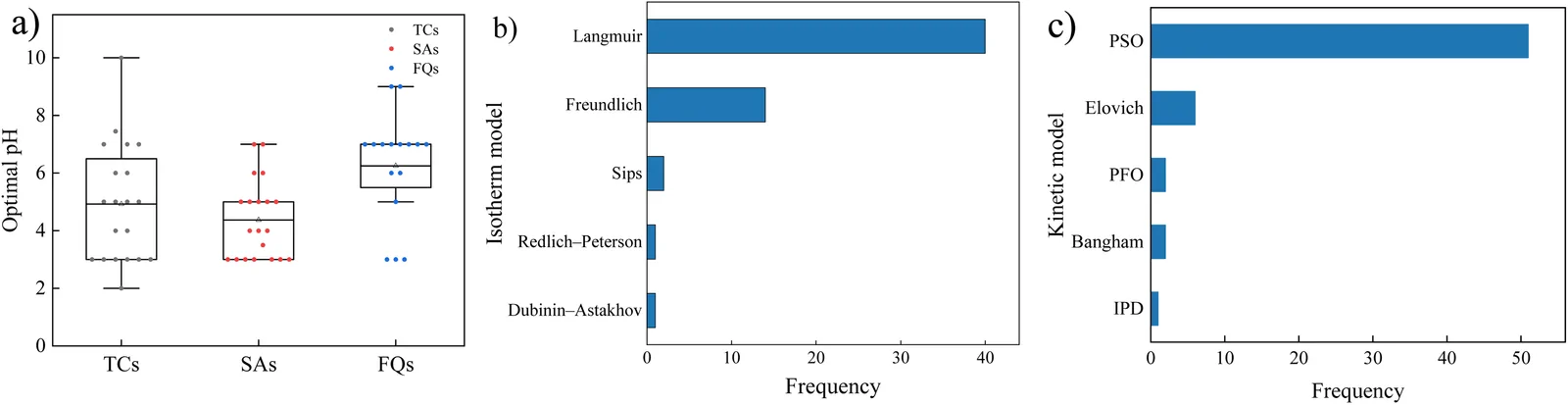
Key factors influencing antibiotic adsorption by modified biochars: (a) optimal pH, (b) isotherm models, and (c) kinetic models.

Antibiotic adsorption by modified biochars is controlled by the coupled effects of solution chemistry, antibiotic speciation, biochar surface charge, pore accessibility, and adsorption-site heterogeneity.^90,91^ Among these factors, pH is the most frequently investigated variable because it simultaneously regulates the ionization state of antibiotics and the protonation/deprotonation of biochar surface functional groups. However, pH alone cannot explain adsorption behavior unless it is interpreted together with antibiotic p*K*_a_ values and the point of zero charge of biochar. Equilibrium and kinetic models provide complementary descriptions of how adsorption is expressed at equilibrium and over time, but they should not be treated as direct evidence for individual molecular mechanisms. As summarized in Fig. 5 and Table S2, the optimal pH distributions, isotherm models, and kinetic models reveal that adsorption performance is not determined by a single factor, but by the compatibility between antibiotic molecular form and the physicochemical properties of modified biochars. The molecular interaction pathways themselves are defined in Section 2 and are therefore not re-described here.

### 5.1. pH-regulated speciation and surface-charge matching

Fig. 5a shows clear class-dependent pH preferences. TCs display a relatively broad optimal pH distribution, ranging from acidic to neutral and even weakly alkaline conditions. Most SA systems are concentrated under acidic to near-neutral conditions, particularly around pH 3–5. FQs are mainly favored near neutral conditions, although several systems show optimal adsorption under acidic or weakly alkaline pH. This distribution indicates that the effect of pH is not universal, but depends on the dissociation behavior of each antibiotic class and the surface charge of the modified biochar.

For TCs, the broad optimal pH window is related to their amphoteric structure and multiple functional groups.^92^ Tetracycline molecules contain phenolic hydroxyl, carbonyl, amide, and dimethylamino groups, allowing them to exist as cationic, zwitterionic, or anionic species under different pH conditions.^43^ As pH changes, the balance between TC molecular charge and biochar surface charge also changes continuously. The persistence of effective adsorption in some TC systems over a relatively broad pH range therefore indicates that unfavorable electrostatic conditions do not necessarily eliminate adsorption when other accessible interfacial domains remain available. This explains why some TC systems in Table S2 maintain effective adsorption over wide pH ranges rather than showing a single narrow optimum.

SAs show a more acidic adsorption preference. Sulfonamide antibiotics are weak acids and become increasingly anionic when pH exceeds their second dissociation constant.^93^ Because many modified biochars also become negatively charged above their pH_pzc_, alkaline conditions usually intensify electrostatic repulsion between anionic SA species and deprotonated biochar surfaces.^94^ Under acidic to near-neutral conditions, this charge incompatibility is reduced.^95^ Therefore, the predominance of acidic optimal pH values for SAs in Fig. 5a reflects a charge-compatibility effect rather than a simple “acidic pH is better” rule. This is particularly important for sulfamethoxazole and sulfadiazine, whose adsorption strongly depends on the balance between molecular ionization and biochar surface charge.

FQs show a different pattern. They contain both carboxyl and piperazinyl or other nitrogen-containing groups and can shift among cationic, zwitterionic, and anionic forms.^27^ Near neutral pH, zwitterionic FQs often show favorable orientation at the biochar–water interface. This is consistent with the frequent optimum around pH 6–7, although acidic or alkaline optima can also occur when the surface chemistry, mineral phases, or charge characteristics of the modified biochar differ.^84^ Thus, the strong material dependence of FQ adsorption identified in Section 4 is also reflected in its pH response.

The pH_pzc_ of modified biochar further determines whether electrostatic attraction or repulsion is favored. Acid activation and oxidation generally introduce –COOH, –OH, C=O, and phosphate-containing groups, which may lower pH_pzc_ and increase surface polarity.^51^ Alkali activation may increase surface basicity, ash content, and negatively charged sites after deprotonation.^38^ Metal and magnetic modifications introduce Fe, Co, Mn, Zn, La, or other metal centers, which can shift pH_pzc_.^96^ Accordingly, adsorption studies should report solution pH together with antibiotic p*K*_a_ and adsorbent pH_pzc_ whenever possible. Importantly, pH should be treated as a regulatory factor rather than as an independent adsorption mechanism. It changes antibiotic speciation, surface charge, and molecular access to the interaction pathways summarized in Fig. 1; changes in adsorption capacity should therefore not be assigned to a specific interaction solely from pH-response curves.

### 5.2. Isotherm and kinetic model distributions

Fig. 5b shows that Langmuir was the dominant isotherm model, appearing 40 times, followed by Freundlich 14 times, Sips twice, and Dubinin–Astakhov and Redlich–Peterson once each. The prevalence of Langmuir fitting suggests that many modified biochars were interpreted as having finite high-affinity adsorption sites and apparent monolayer adsorption behavior.^97^ However, Langmuir fitting should not be overinterpreted as proof of a homogeneous surface. Modified biochars are intrinsically heterogeneous materials containing micropores, mesopores, aromatic domains, mineral phases, oxygen-containing functional groups, defects, and metal oxides. Therefore, Langmuir dominance may partly reflect the selected concentration range and model preference rather than true surface uniformity.^98^

Freundlich fitting, observed 14 times, provides complementary information. It indicates heterogeneous adsorption energies, multilayer tendency, or the coexistence of several adsorption domains.^99^ This is consistent with the actual structure of modified biochars, especially hybrid composites, metal-loaded biochars, and chemically activated materials. The relative success of Freundlich or other heterogeneous models should therefore be interpreted as a description of equilibrium heterogeneity rather than as identification of a specific molecular interaction. The occurrence of Sips, Dubinin–Astakhov, and Redlich–Peterson models, although rare, also suggests that some systems cannot be adequately described by a simple monolayer or empirical heterogeneous model.^100–104^ Therefore, future studies should avoid selecting models only by *R*^2^ and should report nonlinear fitting parameters, residual analysis, error functions, and concentration ranges.

Fig. 5c shows that pseudo-second-order kinetics dominated overwhelmingly, appearing 51 times. This indicates that the PSO equation provides a useful empirical description of the temporal uptake profiles reported for many modified-biochar systems. Nevertheless, PSO fitting does not automatically prove chemisorption. In porous biochars, the apparent kinetic curve may integrate several steps: external film diffusion, rapid occupation of surface sites, intraparticle diffusion, pore filling, and slower stabilization.^105^ Therefore, PSO should be interpreted as an empirical indicator of adsorption kinetics rather than direct evidence of chemical bonding.^106^

The less frequent kinetic models are still meaningful. Elovich fitting, which appeared six times, implies heterogeneous surfaces and changing activation energy during adsorption.^107^ Bangham and intraparticle-diffusion-related models suggest that pore diffusion may contribute to the rate-limiting process in some highly porous biochars.^29^ These approaches are most informative when used alongside, rather than as substitutes for, the dominant PSO fit because they help distinguish mass-transfer limitations from overall uptake-rate description. More generally, kinetic interpretation should distinguish between rate description and mechanism identification. A fitted kinetic equation describes how rapidly adsorption progresses under the tested conditions, whereas evidence for specific molecular interactions should be based on the physicochemical and spectroscopic evidence discussed in Section 2.

Overall, the factors in Fig. 5 should be interpreted as an integrated framework. pH, p*K*_a_, and pH_pzc_ regulate antibiotic speciation and charge compatibility; isotherm models describe the concentration dependence of equilibrium uptake; and kinetic models describe the temporal evolution of adsorption and mass transfer. None of these parameters alone provides definitive evidence for a molecular adsorption mechanism. For cross-study comparison, adsorption data should therefore be evaluated together with pH, pH_pzc_, p*K*_a_, ionic strength, initial concentration, model type, fitting method, and contact time. Without these parameters, *Q*_max_ and kinetic constants may be misleading. Future work should place more emphasis on standardized pH reporting, nonlinear model fitting, diffusion analysis, and environmentally relevant water chemistry, so that the adsorption behavior of modified biochars can be compared more reliably across antibiotic classes and material types.^87^

## 6. Practical implications and application readiness

Although modified biochars have shown promising adsorption capacities for tetracyclines, sulfonamides, and fluoroquinolones, the key question is no longer whether they can adsorb antibiotics under optimized laboratory conditions, but whether they can maintain sufficient performance, safety, and economic feasibility in real treatment systems. As summarized in Table 3, practical application requires a shift from “capacity-oriented material design” to “application-oriented system evaluation”. Therefore, *Q*_max_ should be regarded as only the first screening indicator, whereas real-water performance, regeneration stability, leaching risk, preparation cost, process scalability, and life-cycle impacts should jointly determine the engineering potential of modified biochars.^88,108^ Accordingly, this section treats application readiness as a distinct evaluation dimension that complements the mechanistic framework in Section 2, the material-level analysis in Section 3, the class-resolved performance comparison in Section 4, and the process-factor analysis in Section 5.

**Table 3:** Practical challenges and future research priorities for modified biochars in antibiotic adsorption^a^

Practical aspect	Current observations	Main limitations	Key indicators to report	Future research priorities
Real wastewater matrices	Most reported studies are still conducted in synthetic aqueous solutions or single-antibiotic systems. Real wastewater validation remains limited compared with batch adsorption tests in simplified media	Adsorption performance may be overestimated because real wastewater contains dissolved organic matter, inorganic ions, suspended solids, competing pollutants, and fluctuating pH	Water source, matrix composition, ionic strength, DOM/HA concentration, initial antibiotic concentration, pH, removal efficiency in real water, and comparison with ultrapure water	Future studies should evaluate modified biochars in hospital wastewater, livestock wastewater, aquaculture effluent, municipal wastewater, and multi-antibiotic systems to verify matrix resistance and practical robustness
Regeneration and reusability	Many modified biochars show acceptable adsorption retention after repeated cycles, commonly within 3–5 adsorption–desorption cycles. Some materials retain high removal efficiency, whereas others show obvious capacity decay	Regeneration protocols vary greatly among studies. Harsh regeneration may destroy pore structures, remove surface functional groups, or cause loss of active sites. Some studies report only removal efficiency rather than adsorption capacity retention	Regeneration method, eluent type, number of cycles, final-cycle *Q*_max_ or qe, retention percentage, mass loss, structural changes after regeneration, and regeneration cost	Mild, low-cost, and environmentally friendly regeneration methods should be developed. Future work should report both adsorption performance and structural stability after repeated cycles
Adsorbent recovery and separation	Magnetic or metal-containing biochars can improve solid–liquid separation and facilitate reuse, especially in batch systems	Magnetic loading may block pores, reduce accessible surface area, or cause partial metal release. Quantitative recovery efficiency is rarely reported	Magnetic saturation, recovery efficiency, separation time, particle loss, BET change after reuse, and adsorption performance after magnetic recovery	Future material design should balance adsorption capacity, magnetic recoverability, and structural stability, especially for continuous-flow and fixed-bed applications
Secondary pollution risks	Metal-, mineral-, ionic-liquid-, and polymer-modified biochars can introduce additional active sites and improve adsorption performance through complexation, electrostatic interaction, and functional group enrichment	Potential leaching of Fe, Co, Zn, Mn, La, B, or organic modifiers may cause secondary pollution, especially under acidic conditions or in complex wastewater matrices	Metal leaching concentration, modifier stability, solution pH after adsorption, dissolved organic carbon release, toxicity assessment, and disposal route of spent adsorbents	Long-term leaching tests and ecotoxicity assessments should become routine, particularly for metal-loaded, magnetic, and hybrid composite biochars
Cost and preparation complexity	Conventional activation methods such as KOH, NaOH, H_3_PO_4_, and ZnCl_2_ are widely used, while advanced composites such as MOF-, ionic-liquid-, and polymer-modified biochars provide more functional interfaces	Advanced modification often requires expensive reagents, multi-step synthesis, high pyrolysis temperature, intensive washing, or complicated post-treatment. This may limit large-scale application	Feedstock cost, modifier dosage, pyrolysis temperature, energy input, chemical consumption, washing demand, adsorbent yield, and preparation time	Future studies should prioritize low-cost biomass feedstocks, greener modifiers, simplified synthesis routes, and scalable preparation processes with reduced chemical and energy inputs
Long-term stability and aging behavior	Most studies evaluate fresh adsorbents, while fewer studies examine aged, oxidized, or repeatedly regenerated biochars	Surface oxidation, pore blockage, mineral dissolution, and functional group loss may change adsorption behavior during long-term use	BET surface area before and after use, surface functional groups, zeta potential, pH_pzc_, metal leaching, adsorption capacity after aging, and structural characterization after reuse	Long-term aging tests under realistic pH, ionic strength, light exposure, and wastewater conditions are needed to assess durability and environmental stability
Scale-up and process integration	Most current studies remain at batch scale. Fixed-bed, column, pilot-scale, and long-term continuous-flow studies are still insufficient	Batch adsorption capacity cannot fully reflect hydraulic residence time, pressure drop, breakthrough behavior, fouling, or regeneration feasibility in real treatment systems	Breakthrough curve, bed volume, flow rate, hydraulic residence time, pressure drop, column regeneration efficiency, and long-term operation stability	More fixed-bed, continuous-flow, and pilot-scale studies should be conducted. Modified biochars should also be integrated with biological treatment, membrane filtration, or advanced oxidation as polishing units
Standardized performance evaluation	*Q*_max_, optimal pH, isotherm models, kinetic models, and regeneration data are frequently reported, but experimental conditions differ substantially among studies	Direct comparison across studies is difficult because of inconsistent initial concentration, adsorbent dosage, pH, temperature, contact time, and model-fitting methods	Initial concentration, adsorbent dosage, pH, temperature, equilibrium time, fitting equation, error analysis, model parameters, real-water verification, and regeneration conditions	A standardized reporting framework is needed to improve cross-study comparability and support rational screening of modified biochars for antibiotic adsorption

a This table summarizes the practical challenges and future research priorities for modified biochars in antibiotic adsorption based on the compiled dataset in Table S2 and the representative cases summarized in Table 1. DOM, dissolved organic matter; HA, humic acid; BET, Brunauer–Emmett–Teller surface area; pH_pzc_, point of zero charge; *Q*_max_, maximum adsorption capacity.

### 6.1. Real wastewater matrices and matrix resistance

Most studies summarized in Table S2 were conducted in synthetic aqueous solutions or single-antibiotic systems under controlled pH, contact time, and adsorbent dosage. These experiments are useful for identifying intrinsic adsorption mechanisms, but they cannot fully represent real wastewater. Hospital wastewater, livestock wastewater, aquaculture effluent, pharmaceutical wastewater, and municipal secondary effluent contain dissolved organic matter, inorganic ions, nutrients, suspended particles, surfactants, and multiple coexisting organic pollutants. These matrix components may compete for adsorption sites, block pores, screen electrostatic interactions, alter antibiotic speciation, or form antibiotic–metal/DOM complexes.^103^

Matrix components can reduce adsorption by altering antibiotic speciation, screening surface charge, blocking accessible pores, competing for adsorption domains, or changing the availability of metal-containing surface sites. These effects should be interpreted in relation to the interaction pathways established in Section 2 rather than as independent adsorption mechanisms. Therefore, high *Q*_max_ values measured in ultrapure water should not be directly interpreted as treatment efficiency in practical systems. Future studies should report water source, matrix composition, ionic strength, DOM or humic acid concentration, coexisting ions, initial antibiotic concentration, pH, and the performance difference between ultrapure water and real wastewater. More importantly, multi-antibiotic systems should be introduced because TCs, SAs, and FQs may compete for aromatic domains, polar sites, and metal centers.

### 6.2. Regeneration, reusability, and adsorbent recovery

Regeneration determines whether modified biochar can function as a reusable adsorbent rather than a single-use material. Table S2 shows that many studies evaluated 3–5 adsorption–desorption cycles, using thermal treatment, NaOH elution, ethanol or methanol washing, H_2_O_2_ oxidation, or combined regeneration methods. Some materials retained high performance after repeated use, such as KOH-activated Enteromorpha biochar, ZnCl_2_-activated wheat-straw biochar, and KOH/melamine-doped lignin biochar.^26,42,49^ However, other composites showed obvious capacity loss, indicating that regeneration depends strongly on the reversibility of adsorbate–surface interactions and material stability.^79,109^

Regeneration efficiency depends partly on the reversibility and strength of the interfacial interactions established during adsorption. Stronger or less reversible binding can require harsher desorption conditions, which may in turn damage pore structures, alter surface functionalities, dissolve mineral phases, or accelerate modifier leaching.^16,19^ Therefore, regeneration should not be evaluated only by removal efficiency. Future work should report the eluent type, regeneration method, number of cycles, final-cycle qe or *Q*_max_, capacity retention percentage, adsorbent mass loss, changes in BET surface area, changes in surface functional groups, and regeneration cost.^19^

Adsorbent recovery is another practical issue. Magnetic biochars are attractive because they can simplify solid–liquid separation, but magnetic loading may also block pores, reduce accessible surface area, or release metal species. Quantitative recovery efficiency is rarely reported. Future studies should include magnetic saturation, recovery percentage, separation time, particle loss, adsorption performance after magnetic recovery, and feasibility in continuous-flow or fixed-bed systems.

### 6.3. Secondary pollution risks and long-term stability

The engineered components introduced through the modification strategies discussed in Section 3 may improve adsorption performance but can also create secondary-pollution risks. Metal-containing biochars may release Fe, Co, Zn, Mn, La, B, or other elements under acidic pH, high ionic strength, or DOM-rich conditions.^6,15^ Hybrid composites may release organic modifiers, dissolved organic carbon, fine particles, or residual crosslinking agents.^67,110^ Therefore, the environmental safety of modified biochars cannot be assumed only from adsorption capacity.

Leaching tests should become a routine part of performance evaluation, especially for metal/magnetic and hybrid biochars. Recommended indicators include metal leaching concentration, organic modifier release, dissolved organic carbon, pH variation after adsorption, acute toxicity of treated water, and disposal routes for spent adsorbents. Long-term aging also deserves more attention. In real water, modified biochars may experience oxidation, mineral dissolution, pore blockage, biofilm growth, surface fouling, or loss of functional groups. These changes can alter pH_pzc_, zeta potential, surface polarity, and adsorption capacity.^111^ Therefore, future studies should compare fresh, regenerated, and aged biochars under realistic pH, light exposure, ionic strength, and wastewater conditions.

### 6.4. Cost, scale-up, and life-cycle assessment

For engineering translation, modified biochars should be assessed not only by adsorption performance but also by preparation complexity and life-cycle burden. Highly activated or heteroatom-doped biochars may achieve excellent *Q*_max_, but they often require high pyrolysis temperatures, strong activators, large chemical dosages, acid washing, repeated rinsing, or multi-step synthesis.^4,15^ MOF-derived, ionic-liquid-functionalized, polymer-coated, and metal-doped composites may provide multifunctional interfaces, but their reagent cost, wastewater generation, and synthesis complexity may weaken the low-cost advantage of biochar.^112^

Therefore, future research should integrate adsorption experiments with techno-economic analysis and life-cycle assessment. Key indicators include feedstock availability, transportation distance, pyrolysis temperature, energy consumption, modifier dosage, washing demand, adsorbent yield, regeneration cost, spent-adsorbent disposal, and potential carbon benefits. In addition, most current studies remain at batch scale. Batch *Q*_max_ cannot reflect breakthrough behavior, hydraulic residence time, pressure drop, fouling, particle loss, or column regeneration efficiency. Fixed-bed, continuous-flow, pilot-scale, and long-term operation studies are therefore necessary. Modified biochars should also be evaluated as polishing units integrated with biological treatment, membrane filtration, constructed wetlands, or advanced oxidation.

Overall, the most promising modified biochars are not necessarily those with the highest *Q*_max_, but those that combine adequate adsorption capacity, matrix resistance, regeneration stability, low leaching risk, simple preparation, low cost, and scalable operation. Application readiness should therefore be evaluated independently from laboratory capacity and should not be inferred from *Q*_max_ or mechanistic assignment alone. A standardized reporting framework covering real-water validation, regeneration, leaching, cost, column performance, and life-cycle indicators is essential for moving modified biochars from laboratory adsorption studies to practical antibiotic-contaminated wastewater treatment.

## 7. Conclusions

This review systematically summarized recent advances in modified biochars for the adsorptive removal of tetracyclines, sulfonamides, and fluoroquinolones from aqueous environments. Across the five modification strategies considered, adsorption enhancement is better understood as the coordinated regulation of pore accessibility, surface chemistry, electronic properties, and chemically differentiated interfacial sites rather than as an intrinsic advantage of any single modification route. The adsorption performance of modified biochars showed clear antibiotic-class dependence. TCs exhibited the strongest upper performance envelope, SAs the widest dispersion, and FQs pronounced material dependence. Because the median *Q*_max_ values of the three classes were relatively similar, cross-study capacities should be interpreted as performance envelopes rather than absolute rankings.

Mechanistically, the compiled evidence supports a progressive interfacial framework in which pore accessibility governs molecular entry, electrostatic conditions regulate approach and orientation, molecular-recognition interactions stabilize adsorption, and coordination can provide site-specific anchoring in suitable metal-containing systems. The mechanism-reporting matrix further shows that the prevalence of reported interaction pathways varies with both modification strategy and antibiotic class. However, reporting frequency does not indicate energetic contribution, and equilibrium or kinetic model fitting alone should not be regarded as definitive molecular evidence.

Overall, effective adsorbent development requires integration of antibiotic class, material modification, interfacial compatibility, adsorption performance, and application readiness. However, high adsorption capacity alone is insufficient to determine engineering applicability. Future research should move from capacity-oriented material design toward application-oriented system evaluation, with greater emphasis on real-water performance, regeneration stability, leaching control, continuous-flow operation, scalable preparation, and life-cycle sustainability. The most promising modified biochars are expected to be those that combine adequate adsorption capacity, matrix resistance, reusability, environmental safety, low cost, and practical scalability for antibiotic-contaminated water treatment.

## Conflicts of interest

The authors declare that they have no known competing financial interests or personal relationships that could have appeared to influence the work reported in this paper.

## Supplementary Material

RA-016-D6RA06945B-s001

## Data Availability

No new experimental data were generated in this study. All data analysed in this review were obtained from the published literature cited in the manuscript. The compiled data supporting the adsorption-capacity, optimal-pH, isotherm, kinetic, and mechanistic analyses are provided in the main text, figures, tables, and the supplementary information (SI), particularly Table S2. The comparison of recent review literature used to establish the positioning of this review is provided in Table S1. Supplementary information: details of the literature search, study selection, and data-extraction procedures. See DOI: https://doi.org/10.1039/d6ra06945b.
