# Supplementary material for: Recent advances in modified biochars for the adsorptive removal of typical antibiotics from water: a comparative review of performance, influencing factors, and underlying mechanisms
Source: RSC Adv. 2026 Sep 1;16(42):49616–34. doi: 10.1039/d6ra06945b (PMC13532671; doi:10.1039/d6ra06945b)
Supplement: Supplementary file 1 [file RA-016-D6RA06945B-s001.pdf]

Supplementary Information (SI) for RSC Advances.
This journal is © The Royal Society of Chemistry 2026

Section S1. Literature search, study selection, and data extraction

S1.1. Primary-study search strategy

The quantitative evidence base of this review was established using primary studies retrieved from Web of Science, ScienceDirect, and Google Scholar. The literature search mainly covered studies published from January 2020 to February 2026, and the final search for primary studies included in the quantitative dataset was completed in March 2026. Earlier foundational publications were additionally considered when necessary to support the discussion of biochar physicochemical properties, antibiotic speciation, adsorption models, and adsorption mechanisms.

The search strategy used combinations of terms related to biochar materials, modification routes, target antibiotics, and adsorption processes. Representative search terms included "biochar", "modified biochar", "engineered biochar", "activated biochar", "magnetic biochar", "heteroatom-doped biochar", "antibiotic adsorption", "tetracycline", "sulfonamide", "sulfamethoxazole", "sulfadiazine", "fluoroquinolone", "ciprofloxacin", "levofloxacin", and "wastewater treatment". Additional potentially eligible studies were identified through backward reference screening of relevant primary articles and review papers.

The March 2026 primary-study search defined the dataset used for all quantitative analyses in the present review, including the distributions of modification strategies, adsorption capacities, optimal pH values, isotherm models, kinetic models, and dominant adsorption mechanisms.

S1.2. Supplementary search of recent review literature

To ensure that the revised manuscript was positioned against the most recent review literature, an additional targeted search for review articles was conducted during revision in August 2026. This supplementary search was performed using Web of Science, ScienceDirect, and Google Scholar and focused on recent reviews concerning biochar-based antibiotic remediation.

Search combinations included terms such as (“biochar” OR “modified biochar” OR “engineered biochar” OR “functionalized biochar” OR “magnetic biochar”) AND (“antibiotic” OR “tetracycline” OR “sulfonamide” OR “fluoroquinolone”) AND (“review” OR “critical review” OR “systematic review”). Priority was given to recent reviews published from 2023 to August 2026 that substantially overlapped with the scope of antibiotic adsorption or remediation using pristine, modified, functionalized, magnetic, engineered, or composite biochars.

The identified reviews were evaluated according to their material scope, pollutant scope, treatment pathway, mechanistic emphasis, and application focus. This supplementary review-literature search was used exclusively to update the conceptual positioning of the manuscript, identify overlaps and remaining knowledge gaps, and compare the present review with the most relevant recent reviews. These review articles were not included as data entries in the quantitative adsorption dataset, and therefore did not alter the primary-study dataset established in March 2026.

S1.3. Study screening and eligibility criteria

Records retrieved from the primary-study search and reference-list screening

were first checked to remove duplicate records. The remaining publications were screened in two sequential stages. First, titles and abstracts were examined to exclude studies that were clearly unrelated to modified biochars, aqueous antibiotic adsorption, or the target antibiotic classes. Second, potentially eligible articles were evaluated by full-text reading to confirm the adsorbent type, target antibiotic, adsorption conditions, availability of quantitative adsorption data, and whether adsorption could be clearly distinguished from catalytic degradation when both processes were investigated.

Primary studies were included when they met the following criteria:

1. The adsorbent was a modified or engineered biochar derived from biomass or waste biomass;

2. The target antibiotic belonged to the tetracycline (TC), sulfonamide (SA), or fluoroquinolone (FQ) class;

3. Adsorption experiments were conducted in an aqueous system;

4. Quantitative adsorption performance was reported, particularly maximum adsorption capacity (Qmax) or equilibrium adsorption capacity; and

5. Sufficient information was available on material preparation, adsorption conditions, physicochemical characteristics, or adsorption mechanisms to support comparative analysis.

Studies were excluded when they focused exclusively on pristine biochar, non-biochar carbonaceous materials, gas-phase adsorption, or catalytic degradation without clearly separable adsorption data. Studies lacking quantitative adsorption parameters or providing insufficient information to identify the adsorption system

were also excluded.

When one publication investigated multiple antibiotics or multiple modified biochars, each distinct antibiotic–adsorbent system was treated as an individual data entry for class-resolved analysis. Repeated results originating from the same material–antibiotic system were checked to avoid duplicate counting.

S1.4. Data extraction and comparative analysis

For each eligible primary study, the following information was extracted where available:

- feedstock type;
- modification strategy;
- representative modifier or functional component;
- target antibiotic;
- antibiotic class;
- pyrolysis or preparation conditions;
- Brunauer–Emmett–Teller (BET) specific surface area;
- optimal adsorption pH;
- maximum adsorption capacity (Qmax);
- best-fitting or representative adsorption isotherm model;
- best-fitting or representative kinetic model;
- dominant adsorption mechanisms;
- regeneration or reusability performance; and
- reference source.

Based on the principal modification route, the modified biochars were classified into five categories: alkali activation, acid and salt activation, metal and magnetic composites, heteroatom doping, and hybrid advanced composites. This classification was used to compare how different modification strategies influence pore architecture, accessible surface area, surface charge, functional groups, electronic properties, coordination-active sites, and adsorbent recoverability.

Because adsorption capacity is strongly affected by experimental conditions—including initial antibiotic concentration, adsorbent dosage, solution pH, contact time, temperature, ionic strength, and the concentration range used for isotherm fitting—reported Qmax values were interpreted as performance distributions or performance envelopes rather than absolute rankings of adsorbent superiority. The comparative analysis therefore emphasized class-dependent trends, capacity distributions, modification-related differences, and mechanistic patterns instead of direct one-to-one ranking among materials reported under different experimental conditions.

A total of 50 primary publications were retained in the quantitative evidence base. When one publication reported more than one eligible antibiotic–adsorbent system, multiple data entries were extracted from that publication. The distribution of the five modification strategies was calculated at the publication level, whereas adsorption-capacity distributions, optimal-pH distributions, and isotherm and kinetic model frequencies were analysed at the antibiotic–adsorbent-entry level.

When more than one isotherm or kinetic model was reported for a given antibiotic–adsorbent entry, each model identified by the original study as the best-

fitting or representative model was included in the model-frequency analysis. Consequently, model-frequency counts represent the occurrence of reported representative models rather than the number of unique publications.

The compiled dataset formed the basis for the quantitative comparisons presented in the main manuscript and in Table S2, including adsorption-capacity distributions, class-dependent pH behaviour, model distributions, dominant interaction pathways, regeneration performance, and potential secondary-pollution risks.

Table S1. Comparison of the scope and analytical emphasis of recent reviews on biochar-based antibiotic remediation.

Material scope	Pollutant scope	Removal pathway	Main emphasis	Positioning of the present review	Ref.
Modified biochar in aqueous environments	Antibiotics and antibiotic resistance genes (ARGs)	Primarily adsorption	Mainstream biochar modification methods; comparison of pre- and post-modification adsorption; adsorption mechanisms and influencing factors; benefits, limitations and risks for antibiotics and ARGs.	Restricts the evidence base to antibiotic adsorption by modified/engineered biochars and excludes ARGs from the quantitative dataset; quantitatively compares TCs, SAs and FQs at the antibiotic–adsorbent-system level.	6
Functionalized biochar, with emphasis on physically and chemically modified biochars	Antibiotics and antibiotic residues in water/wastewater	Adsorption	Functionalization routes; changes in pore and surface properties; adsorption mechanisms; effects of operating factors; cost-effectiveness, process economy and environmental implications.	Extends beyond a functionalization-centered overview by comparing five modification families across three antibiotic classes and by interpreting adsorption capacity as class-resolved performance distributions rather than isolated values.	7
Biochar-based systems	Multiple antibiotic classes in liquid streams	Adsorption with discussion of biotic roles	Removal mechanisms organized by antibiotic class; key operating parameters; biochar efficacy; biotic roles; engineering application, prospects and challenges.	Uses a narrower modified-biochar adsorption-only dataset and adds an entry-level quantitative comparison of TCs, SAs and FQs, together with a progressive interfacial framework linking pore access, electrostatic orientation, molecular recognition and site-specific anchoring.	31
Magnetic biochar (MBC)	Multiple antibiotics, including SMX, TC and CIP	Adsorption and catalytic degradation	MBC preparation and modification; antibiotic adsorption and degradation performance; removal mechanisms and influencing factors; magnetic recovery/reuse; preparation cost and secondary-pollution concerns.	Places magnetic biochars within a five-strategy comparison that also includes alkali activation, acid/salt activation, heteroatom doping and hybrid composites; quantitative analysis uses adsorption data only and separately considers leaching, regeneration and application constraints.	25
Biochar-derived adsorbents, from precursor/synthesis to advanced hybrid materials	Antibiotics in wastewater	Predominantly adsorption	Biochar sources and synthesis; activation/modification strategies; operational parameters; adsorption mechanisms; DFT-assisted interpretation; MOF- and MXene-containing hybrid materials; regeneration, risks and future trends.	Adopts a deliberately narrower material/process scope but deeper cross-class quantitative analysis, emphasizing antibiotic–adsorbent-level Qmax distributions, pH-dependent speciation, modification–class relationships and application-readiness indicators.	32
Biochar, modified/engineered biochar and composite materials	Different antibiotics and ARG-related issues in water and soil environments	Adsorption and degradation	Surface chemistry and engineering; adsorption and degradation mechanisms; effects of water/soil chemistry; ARG dynamics; degradation pathways/intermediates; machine learning and pilot-scale prospects.	Focuses specifically on aqueous adsorption by modified/engineered biochars and excludes degradation-only systems from quantitative analysis; integrates five modification families with TC/SA/FQ performance distributions and practical readiness criteria.	33
Modified biochar, including activation, heteroatom/defect engineering and metal integration	Sulfonamide antibiotics (SAs)	Adsorption and degradation	SA-specific structure–performance relationships; structural and electronic regulation; adsorption and catalytic pathways; radical/non-radical mechanisms; bond-selective transformation; stability and engineering scalability.	Broadens the pollutant comparison from SAs to TCs, SAs and FQs while restricting the quantitative evidence to adsorption; highlights how class-dependent molecular size, ionization and functional groups interact with modification chemistry and adsorption mechanisms.	34
Biomass-derived activated carbon and biochar	Tetracycline (TC) as the representative target	Adsorption	Biomass selection; synthesis, activation and surface modification; TC adsorption mechanisms; effects of pH, ionic strength and speciation; kinetics/isotherms; challenges for real-world wastewater applications.	Restricts the adsorbent scope to modified/engineered biochars but expands the antibiotic comparison to TCs, SAs and FQs, enabling cross-class evaluation of modification strategies, performance distributions and application constraints.	35

Note: The comparison describes the principal scope and emphasis of each review rather than implying that topics not listed were completely absent. The fifth column identifies the analytical positioning of the present review. TCs, tetracyclines; SAs, sulfonamides; FQs, fluoroquinolones; ARGs, antibiotic resistance genes; MBC, magnetic biochar; DFT, density functional theory; Q_{max}, maximum adsorption capacity.

Table S2. Summary of modified biochars for antibiotic adsorption. The reference numbers in the Ref. column correspond to the main reference list.

No.	Feedstock	Modification strategy	Representative modifier	Target antibiotic	Antibiotic class	Pyrolysis / preparation condition	BET surface area ($m^2 \cdot g^{-1}$)	Opt. pH	Q_{max} ($mg \cdot g^{-1}$)	Isotherm	Kinetic model	Dominant mechanism	Regeneration / reusability	Ref.
1	Coconut shell	Heteroatom doping	H_3BO_3	Tetracycline	TCs	MW pyrolysis; H_3BO_3/coconut shell = 4:1; 1000 °C; N_2; 1 h	933.39	7	413.223	Langmuir	PSO	π–π	5 cycles; pyrolysis regen.; 80.43% removal retained; 0.76% B leaching	97
2	Corn straw residue	Hybrid advanced composites	Microbial + p-TsOH pre-treatment	Tetracycline	TCs	500 °C carbonization; KOH/NaOH activation; 800 °C; N_2; 60 min	2693	5	1216	Freundlich	PSO/Bangham	PF	>75% after 5 cycles	29
				Sulfadiazine	SAs		2672	Acidic	1083	Freundlich				
3	Highland barley straw	Acid and salt activation	$ZnCl_2$	Tetracycline hydrochloride	TCs	2 M $ZnCl_2$; 750 °C; N_2; 2 h	862.85	3	531.4	Freundlich	PFO/PSO	PF, SC, EA, HB, π–π	NR	99
4	Corn straw	Acid and salt activation	H_3PO_4	Tetracycline	TCs	H_3PO_4/CS = 1:1; 300 °C; N_2; 2 h	463.89	4 (wide 2–10)	227.3	Langmuir	PSO	PF, EA, HB, π–π	4 cycles; Q_e 82→52 $mg \cdot g^{-1}$	92
5	Rice straw	Hybrid advanced composites	$ZnCl_2$ + chitosan	Tetracycline	TCs	BC: 500 °C, 1 h; $ZnCl_2$/BC = 0.45:1; chitosan loading	95.74	10	200.94	Langmuir	PSO	PF, SC, EA, HB, π–π	5 cycles; 57% capacity retained	109
6	Spent coffee ground	Alkali activation	NaOH	Tetracycline	TCs	4 M NaOH; 500 °C; N_2; 2 h	116.59	7.45	113.64	Langmuir	NR	PF, SC, EA, HB, π–π	5 cycles; stable removal	38
7	Tea waste	Metal and magnetic composites	$KHCO_3$ + $FeCl_3$	Tetracycline	TCs	HT 200 °C, 10 h; 700 °C, 2 h	1035.11	3	236.93	Langmuir	Elovich	PF, π–π	NR	107
8	Watermelon rind	Metal and magnetic composites	$FeCl_3$	Tetracycline	TCs	$FeCl_3$/watermelon rind = 2:1; 900 °C; N_2; 2 h	401	3	77.60	Freundlich	PSO	PF, SC, EA, HB, π–π	5 cycles; 83.89% retained	55
9	Rice waste	Metal and magnetic composites	$ZnCl_2$ + $FeCl_3$	Tetracycline	TCs	HTC 230 °C, 3 h; pyrolysis 800 °C, 90 min; N_2	747.76	2	94.63	Langmuir	PSO/IPD	π–π	5 cycles; ~20% decline; low Fe/Zn leaching	77
10	Tea waste	Metal and magnetic composites	$KHCO_3$ + $FeCl_3$	Tetracycline	TCs	HT 200 °C, 9 h; pyrolysis 800 °C, 2 h; N_2	697	3	333.22	Langmuir	PSO	PF, π–π	3 cycles; 125.14→68.21 $mg \cdot g^{-1}$	90
11	Walnut shell	Alkali activation	KOH	Tetracycline	TCs	KOH/WS = 1:1; 900 °C; N_2; 1 h	1713.87	6	607	Langmuir	PSO	PF, EA, HB, π–π	5 cycles; 204.18→169.44 $mg \cdot g^{-1}$; >83% removal	48
12	Rice husk	Metal and magnetic composites	$KMnO_4$	Tetracycline	TCs	500 °C, 2 h; N_2; then 3% $KMnO_4$ (1:20, g/mL), 40 kHz, 60 min, 80 °C, 4 h	24.23	5	266.94	Freundlich	PFO/Elovich	PF	5 cycles; 56.66 $mg \cdot g^{-1}$ retained	57
13	Rice straw	Metal and magnetic composites	$ZnCl_2$, $FeCl_3$	Tetracycline	TCs	3 M $FeCl_3$, 2 h; $ZnCl_2$ addition; 700 °C, 2 h; N_2	1106.87	NR	257.04	Dubinin-Astakhov	PSO	HB, π–π	5 cycles; 94.85% retained (MeOH); H_2O_2 regen. 100%/60 min	101
14	Furfural residue lignin	Heteroatom doping	KOH + Melamine	Tetracycline	TCs	FR lignin extraction; FRLB: 500 °C, 1 h; activation with KOH/melamine (1:5:0.3); 800 °C, 1 h; N_2	3463.87	4	2242	Langmuir	PSO	PF, EA, HB, π–π	5 cycles; 625.3 $mg \cdot g^{-1}$ retained; 93.79% of initial	26
15	Corncob	Metal and magnetic composites	K_2FeO_4	Tetracycline	TCs	Corncob/K_2FeO_4 = 1:1; impregnation 24 h; 800 °C, 2 h; N_2	407.7	3-9	184.62	Langmuir	Elovich	PF, π–π	10 cycles; >91% final efficiency	85
16	Poplar wood powder	Hybrid advanced composites	$[Emim]_2[MnCl_4]$	Tetracycline	TCs	30% KOH pre-activation; 500 °C, 2 h; then $[Emim]_2[MnCl_4]$ (1:15), 800 °C, 2 h	637.33	7	201.5	Langmuir	PSO	PF, HB, π–π	5 cycles; 86% retained; Mn leaching 0.02-0.08 $mg \cdot L^{-1}$	66

No.	Feedstock	Modification strategy	Representative modifier	Target antibiotic	Antibiotic class	Pyrolysis / preparation condition	BET surface area ($m^2 \cdot g^{-1}$)	Opt. pH	Q_{max} ($mg \cdot g^{-1}$)	Isotherm	Kinetic model	Dominant mechanism	Regeneration / reusability	Ref.
17	*Chlorella vulgaris*	Alkali activation	Na_2HPO_4 + KOH	Tetracycline hydrochloride	TCs	Na_2HPO_4 impregnation (0.005 $mol \cdot L^{-1}$, 24 h); KOH/biomass = 0.75:1; 700 °C, 2 h; N_2	1873.78	3	662.91	Langmuir	PSO	π–π	5 cycles; 109.10 $mg \cdot g^{-1}$ after cycle 5; 1 mM NaOH eluent	21
18	Walnut shell	Metal and magnetic composites	$Fe(NO_3)_3$ + $K_2C_2O_4$	Tetracycline	TCs	Pre-carbonization 400 °C, 30 min; BC400/Fe/$K_2C_2O_4$ = 1:1:2; 800 °C, 90 min	1068.67	5	236.9	Freundlich	PSO	π–π	5 cycles; 66.76 $mg \cdot g^{-1}$ retained	96
19	*Enteromorpha prolifera*	Metal and magnetic composites	$Co(NO_3)_2$	Tetracycline hydrochloride	TCs	Co impregnation at 80 °C, 3 h; 900 °C, 2 h; N_2	NR	5	940.4	Langmuir	PSO	SC, EA, π–π	4 cycles; 53.52% removal after cycle 4	74
20	Natural loofah cellulose	Hybrid advanced composites	Polydopamine + ZIF-67	Tetracycline	TCs	PDA coating; ZIF-67 in situ growth; 650 °C, 2 h; N_2	444.59	7	662.25	Langmuir	PSO	PF, EA, HB, π–π	5 cycles; 85.8% retained	30
21	Wheat straw	Acid and salt activation	$ZnCl_2$	Sulfamethoxazole	SAs	$ZnCl_2$ impregnation 6 h; 600 °C, 2 h; N_2	1748.1	5	315.47	Langmuir	PSO	PF, HB	4 cycles; >90% retained	22
22	Pomelo peel	Metal and magnetic composites	$FeCl_3$ + $La(NO_3)_3$	Sulfamethoxazole	SAs	H_3PO_4 activation; 900 °C, 1 h; Fe_3O_4 loading; La doping	1533.4	3	145.7	Langmuir	PSO	PF, EA, HB, π–π	7 cycles; 80.3% retained	93
23	*Pennisetum purpureum*	Metal and magnetic composites	K_2FeO_4	Sulfamethoxazole	SAs	K_2FeO_4 co-pyrolysis; 700 °C, 1 h; N_2	442	3	204.57	Langmuir	PSO	PF, EA, HB, π–π	5 cycles; >60 $mg \cdot g^{-1}$ retained	94
24	Palm fiber	Heteroatom doping	Urea + $FeSO_4$	Sulfamethoxazole	SAs	Fe/urea impregnation (1:1:3); 500 °C, 2 h; N_2	318.203	5	42.9	Langmuir	Elovich/PSO	PF, SC, EA, HB, π–π	4 cycles; 93.4% retained	40
				Sulfadiazine				6	294.12					
25	Coconut shell	Metal and magnetic composites	KOH + $FeCl_3$	Sulfamethazine	SAs	KOH act. (2.5:1); $FeCl_3$ 50 mM; 700 °C 1 h; N_2	1267.3	6	400	Langmuir	PSO	EA, HB, π–π	3 cycles; >80% retained	41
				Sulfamethoxazole				5	454.55					
				Sulfamethoxazole				4	414.2				4 cycles; 377.3→197.2 $mg \cdot g^{-1}$	
26	Bagasse	Metal and magnetic composites	$(NH_4)_2Fe(SO_4)_2$		SAs	BC-550; ball mill with Fe salt (3:2, 400 rpm, 10 min); 800 °C, 60 min; N_2	535.9			Langmuir	PSO	PF, EA, HB, π–π		91
				Sulfamerazine				3	386.3				NR	
				Sulfadiazine					4.18		Elovich			
27	Wood chips	Acid and salt activation	Phosphogypsum		SAs	Phosphogypsum 70 wt%; 450 °C; 30 min	NR	3		Langmuir		EA, HB, π–π, HI	NR	78
				Sulfamethazine					8.91		PSO/Elovich			

No.	Feedstock	Modification strategy	Representative modifier	Target antibiotic	Antibiotic class	Pyrolysis / preparation condition	BET surface area ($m^2 \cdot g^{-1}$)	Opt. pH	Q_{max} ($mg \cdot g^{-1}$)	Isotherm	Kinetic model	Dominant mechanism	Regeneration / reusability	Ref.
28	Spent coffee grounds	Hybrid advanced composites	Chitosan + glutaraldehyde	Sulfamethoxazole	SAs	10% H_3PO_4 pretreatment, 7 h; 600 °C, 1 h; then chitosan/biochar = 1:1, crosslinked with 3% glutaraldehyde, dried at 80 °C	1.78	7.0	14.73	Langmuir	NR	PF, IX, HB, π–π	NR	67
29	Activated sludge biomass	Acid and salt activation	H_3PO_4	Sulfamethoxazole	SAs	H_3PO_4 modification (~2.6 M); MW pyrolysis 800 W, 8 min; washed to neutral, dried	365	4	45.6	Redlich-Peterson	PSO	HB, π–π	ACN desorption tested in column; quantitative retention NR	100
30	Rice straw	Acid and salt activation	H_3PO_4	Sulfamethoxazole	SAs	20 wt% H_3PO_4, solid/liquid = 1:2, impregnation 12 h; 600 °C, 2 h; N_2	233	3	148.62	Langmuir	PSO	HB	4 cycles; stable removal	51
31	*Enteromorpha prolifera*	Metal and magnetic composites	KOH + $FeSO_4/FeCl_3$	Sulfamethoxazole	SAs	Prepyrolysis 500 °C, 5 h; KOH/NB = 2:1, copyrolysis 800 °C, 2 h; then Fe^{2+}/Fe^{3+} coprecipitation with $NH_3 \cdot H_2O$ at 80 °C, 30 min	1512.53	7	502.2	Freundlich	PSO	PF, EA, HB, π–π	7 cycles; 87.29% retained	23
32	Coffee ground	Alkali activation	KOH + H_3PO_4	Sulfadiazine	SAs	Pyrolysis 700 °C, 1 h; KOH activation (2 eq.) at 700 °C, 1 h; then 35 wt% H_3PO_4 modification (biochar/H_3PO_4 = 1:2), repeated impregnation/sonication, washed to neutral	1365	5	139.2	Langmuir	PSO	π–π, HI	NR	95
33	Sludge	Hybrid advanced composites	Biosurfactant	Sulfamethoxazole	SAs	Sludge dried; pyrolysis 673 K, 240 min; then BC/biosurfactant = 5 g/0.5 g in 100 mL H_2O, 313 K, 24 h	22.42	3	43.61	Freundlich	PSO	PF, π–π	5 cycles; strong decline; ~2.6% removal after cycle 5	79
34	Wheat straw	Acid and salt activation	$ZnCl_2$	Sulfamethoxazole	SAs	$ZnCl_2$/wheat straw = 1:1; impregnation 12 h; 80 °C evaporation; pyrolysis 800 °C, 4 h; N_2; washed with 0.1 M HCl to neutral	1328	4	597.57	Freundlich	PSO	PF, EA, HB, π–π	Batch: 5 cycles, 328.54 $mg \cdot g^{-1}$ retained; column: 3 cycles, 685.18→464.54 $mg \cdot g^{-1}$	42
35	*Enteromorpha prolifera*	Alkali activation	KOH	Sulfamethoxazole	SAs	Pre-pyrolysis 500 °C, 5 h; then biochar/KOH = 1:0.5, 800 °C, 2 h; N_2; HCl and water washing	2172.08	5	744.32	Langmuir	PSO	PF, EA, HB, π–π	5 cycles; 94% retained	49
36	Rice straw	Metal and magnetic composites	Phosphomolybdic acid + $Fe(NO_3)_3$	Tetracycline	TCs	Reflux oxidation with PA (0.2 M) + FN (0.4 M), 120 °C, 4 h; then pyrolysis 700 °C, 1 h; N_2	73.39	3	1781.81	Freundlich	PSO	HB, SC, IX, EA	5 cycles; 197.76→61.69 $mg \cdot g^{-1}$	73
				Ciprofloxacin	FQs				202.68			HB, SC, IX, EA	recyclable; 97.95→65.95 $mg \cdot g^{-1}$ after 1 cycle	
37	Rice husk	Metal and magnetic composites	KOH + Fe_3O_4	Levofloxacin	FQs	KOH activation (BC/KOH = 1:2, 24 h); pyrolysis 800 °C, 1 h, N_2; then one-step hydrothermal Fe_3O_4 loading ($FeCl_3 \cdot 6H_2O$ + CH_3COONa + PEG, 200 °C, 2 h)	73.99	5	57.83	Freundlich	PSO	PF, EA, HB, π–π	5 cycles; 79.66% retained in coupled adsorption-photodegradation system	83
38	Anaerobic digestate	Metal and magnetic composites	$FeCl_3$ + KOH	Ciprofloxacin	FQs	Digestate dried 105 °C, 48 h; pyrolysis 600 °C, 2 h; then sequential modification with 0.1 M $FeCl_3$ and 1 M KOH (solid/liquid = 1:10, 2 h each)	176.11	3	117.93	Langmuir	PSO	SC	3 cycles; removal ~86%→82%→78%	58

No.	Feedstock	Modification strategy	Representative modifier	Target antibiotic	Antibiotic class	Pyrolysis / preparation condition	BET surface area ($m^2\cdot g^{-1}$)	Opt. pH	Q_{max} ($mg\cdot g^{-1}$)	Isotherm	Kinetic model	Dominant mechanism	Regeneration / reusability	Ref.
39	Apple branches	Heteroatom doping	Urea + NH_4F + potassium formate	Norfloxacin	FQs	Two-step pyrolysis: first with potassium formate + urea at 800 °C, 1 h; then secondary fluorination with NH_4F (sample/NH_4F = 1:3) at 600 °C, 1 h	2187	7	552.65	Sips	PSO	PF, EA, HB, π–π	4 cycles; 408 $mg\cdot g^{-1}$ retained; mass recovery 95.23% after one regeneration	27
				Moxifloxacin				6–8	283.32	Langmuir	PSO	EA, HB, π–π	5 cycles; >90% retained	
40	Bamboo powder	Acid and salt activation	$ZnCl_2$	Ciprofloxacin	FQs	Bamboo/$ZnCl_2$ = 1:3; 25 °C impregnation 2 h; pyrolysis 700 °C, 2 h; then 2 M HCl, 65 °C, 6 h	NR	6–8	281.19	Langmuir	PSO	EA, HB, π–π	5 cycles; >90% retained	82
				Ofloxacin				6–8	250.46	Langmuir	PSO	EA, HB, π–π	5 cycles; >90% retained	
41	Cotton husk	Acid and salt activation	H_3PO_4	Ciprofloxacin	FQs	Cotton husk impregnated with 30% H_3PO_4 (15 mL per 10 g biomass), pyrolyzed at 600 °C for 120 min under N_2 (0.1 $L\cdot min^{-1}$), heating rate 5 °C min^{-1}	316.1	6	572.8	Langmuir	PSO	PF, EA, HB, π–π	EtOH regeneration; 3 cycles, 196.96 $mg\cdot g^{-1}$ retained (70.79%)	52
42	Tea waste	Heteroatom doping	Urea + $MnSO_4$	Ciprofloxacin	FQs	Tea waste 6.0 g + urea 6.0 g + $MnSO_4\cdot H_2O$ 4.3 g; impregnated/stirred 10 h; dried at 80 °C for 8 h; pyrolyzed at 500 °C for 2 h	234.54	6	121.42	Freundlich	PSO	HB, π–π	5 cycles; 92.7 $mg\cdot g^{-1}$ retained	65
43	Moss (bryophyte)	Metal and magnetic composites	$FeCl_3$ + $FeCl_2$	Enrofloxacin	FQs	Moss dried at 105 °C for 24 h; pyrolysis at 800 °C for 2 h under N_2 (10 °C$\cdot min^{-1}$); then Fe_3O_4 loaded by co-precipitation with $NH_3\cdot H_2O$, BC:MNP = 2:1	69.26	7	11.62	Langmuir	PSO	PF, EA, HB, π–π, HI	3 cycles; >70% removal retained	86
44	Durian peel	Alkali activation	KOH	Ciprofloxacin	FQs	Durian peel powder/KOH = 1:1; pyrolysis at 700 °C for 2 h under N_2 (150 $mL\cdot min^{-1}$), heating rate 10 °C$\cdot min^{-1}$; then 1 M HCl washing and drying at 60 °C	1484.31	NR	475.5	Langmuir	PSO	PF, HB, π–π	Thermal regeneration best: 72% (cycle 1), 33% (cycle 3)	47
45	Waste peanut meal	Heteroatom doping	Self-N doping + potassium oxalate	Levofloxacin	FQs	Pre-carbonization at 300 °C for 1 h under N_2; then BC/activator = 1:2, pyrolysis at 900 °C for 1 h (5 °C$\cdot min^{-1}$); 2 M HCl washing for 4 h	2119.96	9	775.97	Langmuir	PSO	PF, HB, π–π	Pyrolysis + NaOH regeneration; 4 cycles, 85.93% removal retained	28
46	Longan seed	Heteroatom doping	H_3BO_3 + KOH	Lomefloxacin	FQs	Pre-carbonization with H_3BO_3 at 600 °C for 2 h; then BBC-2/KOH activation at 850 °C for 1 h under N_2 (30 $mL\cdot min^{-1}$), heating rate 3 °C$\cdot min^{-1}$; dried at 70 °C	2657.3	7	1295.4	Langmuir	PSO	π–π	NR	63
47	Delignified bamboo	Metal and magnetic composites	Fe_3O_4 + KOH	Norfloxacin	FQs	Delignification with $NaClO_2$/acetic acid hydrothermal magnetization with $FeCl_3\cdot 6H_2O$ + $FeCl_2\cdot 4H_2O$ at 160 °C for 6 h; pyrolysis at 400 °C for 2 h under N_2; then KOH activation (1:1) at 800 °C	1253.66	3	458.43	Langmuir	PSO	EA, HB, π–π	4 cycles; 62.5% removal retained	24

No.	Feedstock	Modification strategy	Representative modifier	Target antibiotic	Antibiotic class	Pyrolysis / preparation condition	BET surface area ($m^2 \cdot g^{-1}$)	Opt. pH	Q_{max} ($mg \cdot g^{-1}$)	Isotherm	Kinetic model	Dominant mechanism	Regeneration / reusability	Ref.
						for 2 h; 0.1 M HCl washing								
48	Hardboard waste	Metal and magnetic composites	$Fe(NO_3)_3$	Ciprofloxacin	FQs	Impregnation with 0.1 M $Fe(NO_3)_3$ at 100 °C for 2 h; dried at 80 °C for 24 h; pyrolysis at 800 °C for 2 h under N_2, heating rate 10 °C·min^{-1}	530	7	43.27	Sips	NR	HB, π–π	50% EtOH regeneration; 4 cycles, 64.09% removal retained; no Fe leaching	102
49	Cedar bark	Metal and magnetic composites	NaOH + Fe/Co bimetals	Ofloxacin	FQs	N_2 pyrolysis of cedar bark; NaOH activation; then Fe/Co bimetal modification to form FMBC; exact ratio/temperature NR in visible main text	496.42	7	142.19	Langmuir	PSO	PF, EA, HB, π–π, HI	Best at 430 °C thermal regeneration; 5 cycles, 89.31% retained	56
50	Areca nut husk	Metal and magnetic composites	$ZnCl_2$ + $Fe(NO_3)_3$	Enrofloxacin	FQs	Pre-carbonization at 350 °C for 30 min; then BC350/$ZnCl_2$/$Fe(NO_3)_3$·9H2O = 1:2:0.5, stirred 24 h; co-pyrolysis at 650 °C for 90 min under N_2; washed with 0.1 M HCl to neutral	1475.35	9	198.13	Freundlich	PSO	PF, π–π, EA, HB, SC	Methanol + H_2O_2 best; 4 cycles, 61.12 mg·g^{-1} retained	59

Note: NR, not reported; PF, pore filling; SC, surface complexation; EA, electrostatic attraction/interaction; HB, hydrogen bonding; HI, hydrophobic interaction; IX, ion exchange; PFO, pseudo-first-order; PSO, pseudo-second-order; IPD, intraparticle diffusion; ACN, acetonitrile; MeOH, methanol; EtOH, ethanol; MW, microwave; HTC, hydrothermal carbonization.
